# Electrical modulation of transplanted stem cells improves functional recovery in a rodent model of stroke

**DOI:** 10.1038/s41467-022-29017-w

**Published:** 2022-03-15

**Authors:** Byeongtaek Oh, Sruthi Santhanam, Matine Azadian, Vishal Swaminathan, Alex G. Lee, Kelly W. McConnell, Alexa Levinson, Shang Song, Jainith J. Patel, Emily E. Gardner, Paul M. George

**Affiliations:** 1 Department of Neurology and Neurological Sciences, Stanford University School of Medicine, Stanford, CA 94305 USA; 2 Department of Pediatrics, University of California, San Francisco, CA 94305 USA

**Keywords:** Implants, Regeneration and repair in the nervous system, Biomedical engineering, Neural stem cells, Stroke

## Abstract

Stroke is a leading cause of long-term disability worldwide, intensifying the need for effective recovery therapies. Stem cells are a promising stroke therapeutic, but creating ideal conditions for treatment is essential. Here we developed a conductive polymer system for stem cell delivery and electrical modulation in animals. Using this system, electrical modulation of human stem cell transplants improve functional stroke recovery in rodents. Increased endogenous stem cell production corresponds with improved function. Transcriptome analysis identified stanniocalcin 2 (STC2) as one of the genes most significantly upregulated by electrical stimulation. Lentiviral upregulation and downregulation of STC2 in the transplanted stem cells demonstrate that this glycoprotein is an essential mediator in the functional improvements seen with electrical modulation. Moreover, intraventricular administration of recombinant STC2 post-stroke confers functional benefits. In summation, our conductive polymer system enables electrical modulation of stem cells as a potential method to improve recovery and identify important therapeutic targets.

## Abstract (web summary)

Paul George and colleagues developed a conductive polymer system to enable stem cell delivery and electrical modulation in vivo. Employing this system improved functional stroke recovery in rodents and identified important repair pathways.

## Introduction

Of the over 10 million strokes that occur annually, the majority of survivors are left with functional deficits^1^. Early in development the brain is quite plastic^2^, but as the human brain ages, its intrinsic capabilities to recover from insults such as stroke decline^3^. Exogenous cell therapeutics including neural progenitor cell (NPC) delivery have emerged in an attempt to offset stroke-induced neuronal loss and promote functional recovery^4–7^. Alternatively, augmentation of the brain’s intrinsic mechanisms of repair such as endogenous stem cell production has been utilized to enhance restoration following ischemia^8–12^. Effective tools to optimize the convergence of these two approaches may prove beneficial to improving stroke outcomes and create the optimal microenvironment for repair.

Manipulation of the recovering neural environment through bioengineering approaches has largely focused on targeting a single stimulus paradigm. Electrical fields have been tailored to normalize the disrupted neural balance after stroke^13–17^. Biomaterials are able to deliver important chemical factors or optimize stem cell delivery in therapeutic applications, but often lack the ability for continuous modulation after implantation^18–20^. This single modality and static approach for neural rehabilitation juxtaposes the natural process of development, where an intricate combination of chemical, tactile, and electrical signals guide stem cells during brain formation. Conductive polymers offer a platform to continuously modulate cells and tissue across these stimulation modalities^21^. Electrical stimulation of NPCs seeded on conductive polymers alter trophic factor release from NPCs, a primary mechanism of stem cell-enhanced stroke repair^22–24^. For instance in our prior work, we found that the VEGF pathway was augmented when electrically stimulated, and these pre-conditioned cells, when transplanted, enhance recovery after stroke in rodents. However, the system was confined to stimulating the cells in vitro.

In this work, we demonstrate how the unique ability to continuously interact with the neural environment electrically via the conductive polymer provides further opportunity to shape the post-stroke brain to promote functional recovery.

## Results

### Conductive polymer system for in vivo NPC stimulation

**Fig. 1:**
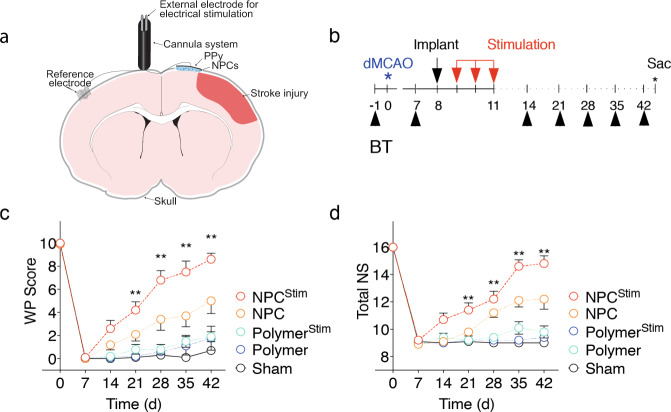
Electrical stimulation of conductive polymer-stem cell implant improves functional stroke recovery. **a** Schematic representation of the transplanted conductive polymer-based cannula system (PPy-polypyrole, NPC – neural progenitor cells). **b** Experimental timeline with training three times in the week prior to dMCA occlusion. Black arrows indicate behavioral testing (BT). Red arrows indicate stimulation after implantation. **c** Vibrissae-forepaw (WP) behavioral testing. There is a statistically significant difference in WP Scores between NPC^Stim^ and Polymer^Stim^, Polymer, and Sham groups at 3 weeks post-stroke, and a significant difference in WP Scores between NPC^Stim^ and all other groups at 4 weeks post-stroke and onward. **d** Total neurological score (NS) testing. NPC^Stim^ exhibited significant difference in NS at individual timepoints 3 weeks post-stroke and beyond, in comparison to the NS of all other groups; moreover, there was a significant difference in NS Scores between NPC^Stim^ and Polymer^Stim^, Polymer, and Sham groups at 3 weeks post-stroke and onward. **c** Analyzed using a log-transform two-way repeated measures ANOVA, which revealed a statistically significant interaction between the effects of treatment group and WP Scores (*F* [24, 270] = 8.379, *P* < 0.0001), followed by Dunnett’s multiple comparisons test. **d** Analyzed using Kruskal–Wallis test (*P* < 0.0001), followed by Dunn’s multiple comparisons test. For both, **P < 0.01, data shown as mean ± SEM, *n* = 10 per group.

We developed a conductive polymer-based system to deliver NPCs and continuously interact with the neural environment to augment stroke repair (Fig. 1a and Supplementary Fig. 1). The conductive polymer system provides the ability to manipulate the electrical environment in vivo and modulate the transplanted NPCs to provide chemical stimulus (via released factors) to the post-stroke brain. Because of the polymeric properties of the implantable system, it offers advantages over traditional inorganic electrodes of forming intimate connections with the NPCs and neural tissue after transplantation^25^. We optimized the conductive polymer system in vitro based on our previous work (Supplementary Fig. 2), where we found that VEGFA was a key trophic factor that gets upregulated with electrical stimulation in vitro^22^. Therefore, we chose the electrical parameters of 800 mV, 100 Hz, and 1 h for the current work as it maximizes VEGFA secretion (Supplementary Notes). To address the impact of electrical stimulation on NPC biology/proliferation, we performed a cell proliferation study with the optimized stimulation parameters 1 h every day for the first 3 days. We found electrical stimulation increased NPC proliferation at day 4 and 7 in culture (Supplementary Fig. 3). Furthermore, the expression of neural stem cell markers such as Nestin and Pax6, remained the same without any differentiation with or without electrical stimulation during this timeframe.

### Electrical stimulation of transplanted NPCs improves functional stroke recovery

To explore the impact of modulating the stem cell and electrical environment on stroke recovery, we implanted this system into rats who had underwent the distal middle cerebral artery occlusion (dMCAO) model of stroke. NPCs delivered via the conductive polymer system were electrically stimulated in vivo (conductive polymer system implantation is 1 week after stroke, electrical stimulation occurs on days 1, 2, and 3 after implantation, Fig. 1b). Animals that received NPCs combined with electrical stimulation (NPC^Stim^) experienced earlier and sustained improved recovery compared to the unstimulated group (NPC) as assessed by the vibrissae-forepaw model (WP) and the neurological severity scale (NSS) (Fig. 1c, d, Supplementary Figs. 4 and 5), both of which are well-characterized assessments of behavioral function in various MCAO stroke models^26,27^. The control groups of conductive polymer alone with or without electrical stimulation (Polymer and Polymer^Stim^ respectively) and the Sham group (no treatment) showed minimal recovery. As expected, the NPC group also outperformed the control groups but to a lesser extent than the NPC^Stim^ condition. No difference was observed in stroke size between the groups (Supplementary Fig. 6 and Supplementary Notes).

### Combined electrical stimulation and NPC transplants upregulate endogenous stem cells

**Fig. 2:**
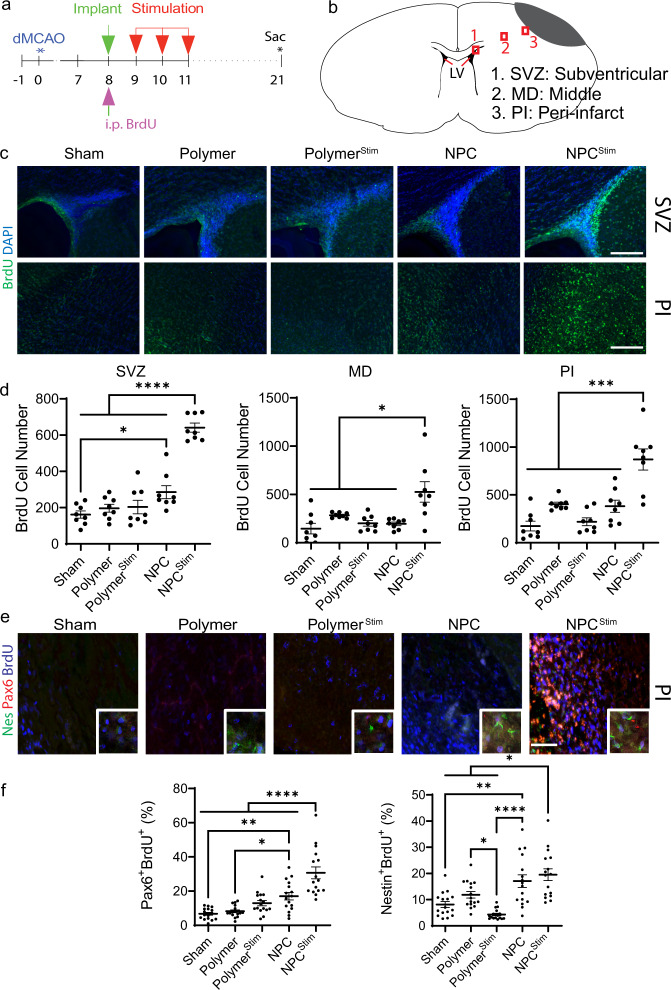
Combined electrical stimulation and NPCs increase endogenous stem cell production. **a** Experimental timeline with electrical stimulation three times after implantation surgery. BrdU was administered i.p. when first stimulation was applied. **b** Schematic of areas of interest in brain tissue. Gray indicates ischemic stroke lesion. Red squares indicate the area of interest: 1. SVZ, 2. MD; and 3. PI (LV – lateral ventricle). **c** Representative images of fluorescent-labeled cells. Green indicates BrdU positive cells, whereas blue indicates cell nucleus. Scale bar indicates 200 µm. **d** BrdU positive cells in certain area of interest. Data are shown as mean ± SEM, *n* = 8 images/4 rats. **e** Representative images of fluorescently labeled cells. Blue indicates BrdU, green indicates Nestin (Nes), and red indicates Pax6 positive cells. Scale bar indicates 200 µm. **f** Co-stained BrdU and Pax6 or Nestin positive cells in peri-infarct area. Data are shown as mean ± SEM, *n* = 16 images/4 rats. **d**, **f** Analyzed using a one-way ANOVA, followed by Tukey’s HSD post-hoc test with *P < 0.05, **P < 0.01, ***P < 0.001, and ****P < 0.0001.

We proposed that the neural environment created through electrical stimulation in tandem with cues generated from the transplanted NPCs enhanced the brain’s intrinsic repair mechanisms such as endogenous stem cell production. The production of endogenous stem cells in the subventricular zone (SVZ) increases after stroke^8,11,28^. These endogenous stem cells travel to the ischemic area and impact recovery. An increase in production correlates to improved recovery, and manipulations to prevent endogenous stem cell production worsens stroke size and functional outcomes^12,29^. Examination of BrdU (5-bromo-2’-deoxyuridine)-positive cells showed an increase in endogenous stem cells across 3 different brain regions after stroke in the NPC^Stim^ group (Fig. 2a–d). The higher numbers of BrdU-labeled cells across all three regions points to likely increased production of NPCs and greater migration of the cells to the peri-infarct tissue in the NPC^Stim^ group. Human nuclear staining (HuNu) delineated the transplanted NPCs from endogenous stem cells and confirmed minimal integration of transplanted cells (~1%) in the host tissue (Supplementary Fig. 7). Secondary staining demonstrated that endogenous stem cells in the peri-infarct region are neural progenitors with the electrically stimulated cells (NPC^Stim^) containing a higher percentage of mature mitotic markers (Pax6 and Nestin, Fig. 2e, f) than all other groups. Co-staining the Nestin+/BrdU+ cells in the peri-infarct region with S100B (astrocyte marker) revealed that there was a minimal cross-reactivity of Nestin with reactive astrocytes (<3%; Supplementary Fig. 8). Moreover, the electrical stimulation does not impact differentiation of several key markers at the 3-week post-stroke time point (Supplementary Fig. 9). The polymer alone, NPC, and NPC^Stim^ appear to increase angiogenesis in the PI area (Supplementary Fig. 9b) similar to prior work with NPCs^22^. Because in vivo electrical stimulation did not further enhance this response, it suggests another mechanism for the functional improvement seen with combined therapy. Furthermore, staining for other types of cells such as the neurons and glia revealed that electrical stimulation does not affect their cell number at peri-infarct and stroke regions (Supplementary Notes and Supplementary Fig. 10). Given prior work demonstrating endogenous stem cells enhance stroke recovery^9–12^, the upregulation of endogenous stem cell production highlights an important potential mechanism through which the NPC^Stim^ group improves stroke recovery.

### Electrical stimulation modifies NPC transcriptome

**Fig. 3:**
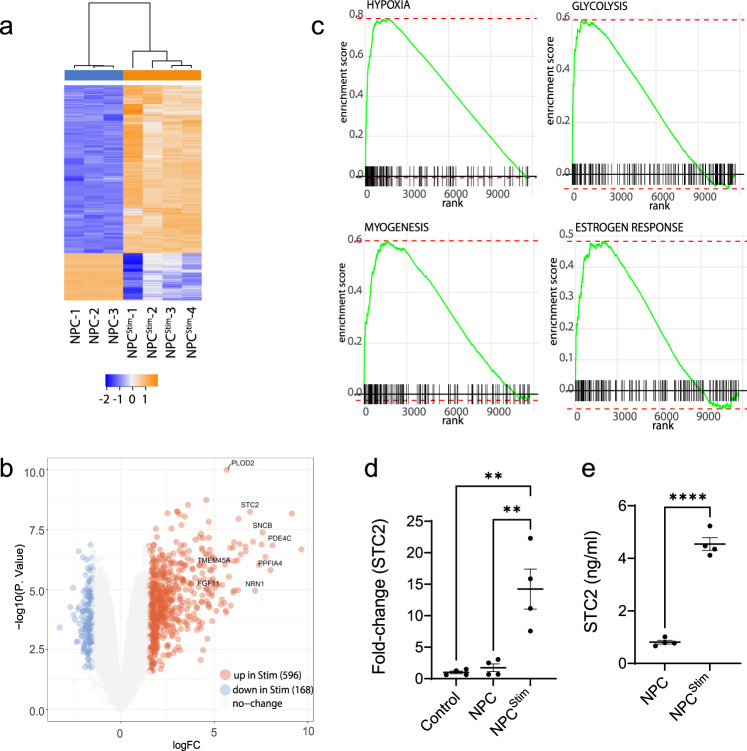
Transcriptome changes between electrically stimulated and unstimulated NPCs. **a** Heatmap of transcriptome changes in NPCs from electrical stimulation (NPC – unstimulated, NPC^Stim^ – electrically stimulated; blue-downregulated, orange-upregulated). **b** Volcano plot demonstrating changes in genes with electrical stimulation (blue-downregulated, orange-upregulated). **c** Top Gene set enrichment pathways with STC2 as the leading edge. **d** qRT-PCR analysis indicated that STC2 in NPCs was upregulated by electrical stimulation. **e** ELISA study indicated that the level of STC2 protein after electrical stimulation (NPC^Stim^) is much higher than that in non-stimulated NPCs (NPC). **d**, **e** Analyzed using a one-way ANOVA, followed by Tukey’s HSD post-hoc test with **P < 0.01, ****P < 0.0001, data shown as mean ± SEM, *n* = 4.

Electrical stimulation alters the properties of stem cells^22,30–32^ To determine how electrical stimulation modified NPCs to improve functional stroke recovery, we performed RNA sequencing (RNAseq) analysis. The transcriptome was compared between NPCs that were electrically stimulated and those that were unstimulated on our conductive polymer scaffold. Cells that were electrically stimulated had a profound transcriptome change as evident from our unsupervised multidimensional scaling (MDS) plot and hierarchical clustering (Supplementary Fig. 11). Notably, electrical stimulation alone produced two distinct clusters from the same NPCs. Furthermore, differential gene expression showed robust differences between the two groups. Even with stringent statistical filters (false discovery rate (FDR) < 0.05, log fold change >1.5), we observed differential gene expression (DGE) with 596 genes upregulated and 168 genes downregulated, when cells were electrically stimulated on the conductive polymer system (Fig. 3a, b). These gene changes include pathways relevant to NPC microenvironments such as glycolysis as well as hypoxia. From our analysis, stanniocalcin-2 (STC2) was identified as an NPC gene that was one of the most significantly modified with electrical stimulation (NPCStim) (*p* = 9.55 × 10^−6^ after adjusting for multiple comparisons and by logFC values, 6.905, Supplementary Table 1).

Gene Set Enrichment Analysis (GSEA) is a widely used pathway analysis to show overrepresentation of a particular gene set with an a priori gene set. Unlike over representation analysis, which normally relies on a hypergeometric distribution to determine significant overlap between two sets of gene; GSEA utilizes all genes ranked by log fold change^33^. For this reason, it is thought to be more accurate particularly with studies with lower sample size or quality since it is not dependent on any predefined statistical cutoff. Here, we implement a fast GSEA in the fgea R package^34^. Genes are first rank by logFC and given a priori defined set of gene S. The algorithm determines whether the members of S are either randomly distributed throughout the ranked gene list (L) or clusters in the top or lower portion. The degree by which set S is represented at the top or lower half is defined with enrichment (ES) score. This score is represented as the curve seen in Fig. 3c and the bottom hash are the genes that are found in set S based along the L logFC ranking. The “leading edge” genes are the genes that contributes most to the enrichment scores. Gene set enrichment analysis (GSEA) showed that 4 of the top 15 hallmark pathways have STC2 as the leading-edge gene (Fig. 3c and Supplementary Table 2). We verified the upregulation of STC2 using qRT-PCR and ELISA analysis (Fig. 3d, e). In addition to STC2, qPCR was used to verify other genes that were among the most significantly upregulated and had biological interest, such as genes involved with secreted proteins (Supplementary Fig. 12). Several candidates were not found to be upregulated with qPCR confirmation (Supplementary Table 3). Given STC2 plays a role in cell proliferation and survival, we found it an interesting candidate to further explore its role in stroke recovery and the increase in endogenous stem cell production seen with combined stem cell delivery and electrical stimulation^35–39^.

### Upregulation of STC2 in NPCs improves functional stroke recovery and effects endogenous stem cell production

To substantiate STC2 in stem cell-enhanced recovery, we utilized lentiviral constructs to knock down (STC2^KD^) or over-express STC2 (STC2^UP^) in the NPCs delivered on our conductive polymer system (Supplementary Fig. 13). Scrambled constructs (Scramble^KD^) served as controls.

**Fig. 4:**
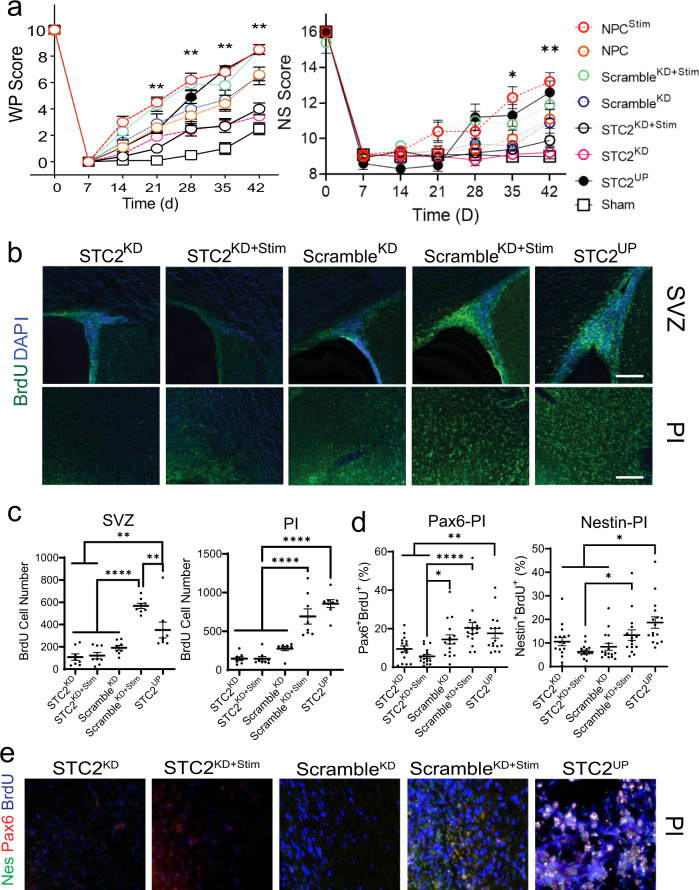
STC2 is integral for improved stroke recovery. **a** Vibrissae-forepaw (WP) and total neurological score (NS). There is a statistically significant difference in NPC^Stim^, STC2^UP^, and Scramble^KD+Stim^ groups versus all other groups starting at week 3 and onward for WP Scores and week 5 and onward for NS scores. STC2^KD^ and STC2^KD+Stim^ showed no significant behavioral recovery at any timepoint in either WP Scores or NS Scores. **b** Representative images of fluorescently labeled cells. Green indicates BrdU positive cells, whereas Blue indicates cell nucleus. Scale bar indicates 200 µm. **c** BrdU positive cells in certain areas of interest. Data are shown as mean ± SEM, *n* = 8 images/4 rats. **d** PAX6/BrdU or Nestin/BrdU positive cells in certain areas of interest. Data are shown as mean ± SEM, *n* = 16 images/4 rats. **e** Representative images of fluorescently labeled cells. Green indicates Nestin positive cells whereas Red indicates PAX6 positive cells. Blue indicates BrdU positive cells. Scale bar indicates 200 µm. **a** Analyzed using a Kruskal–Wallis test followed by post-hoc pairwise Mann–Whitney *U* test with Benjamini–Hochberg correction to control the false discovery rate at the 0.05 level. For both, *P < 0.01, **P < 0.001, data shown as mean ± SEM, *n* = 10 per group. **c**, **d** Analyzed using a one-way ANOVA, followed by Tukey’s HSD post-hoc test with *P < 0.05, **P < 0.01, and ****P < 0.0001.

We determined that increased STC2 through electrical stimulation or lentiviral constructs (NPC^Stim^, Scramble^KD+Stim^, and STC2^UP^ respectively) improves functional recovery (Fig. 4a, Supplementary Figs. 14 and 15). If STC2 levels are reduced via lentiviral knockdown, the effect of electrical stimulation is lost, and recovery is similar to levels observed in Sham animals (STC2^KD^ and STC2^KD+Stim^). With reduced NPC production of STC2, the benefit of the therapy in stimulated and unstimulated groups was diminished, suggesting that STC2 is an important pathway for stem cell effects on stroke recovery irrespective of its role in the improvement seen with electrical stimulation.

To determine if manipulation of the STC2 produced by the NPCs had similar effects on endogenous stem cell production, BrdU staining was performed. We found that only in the upregulated STC2 group and the scrambled electrically stimulated group (STC2^UP^ and Scramble^KD+Stim^ respectively) was an increase in endogenous NPCs with more mature mitotic markers (PAX6 and Nestin) observed (Fig. 4b–e and Supplementary Fig. 16). No change in stroke size was seen in any of the groups (Supplementary Fig. 17). Thus, STC2 appears to be an essential pathway for the increased production of endogenous stem cells in addition to functional recovery following stroke. These experiments demonstrate how the conductive polymer scaffold can elucidate repair pathways in the NPCs to determine important molecules for further research.

### Intraventricular administration of STC2 improves functional stroke recovery

**Fig. 5:**
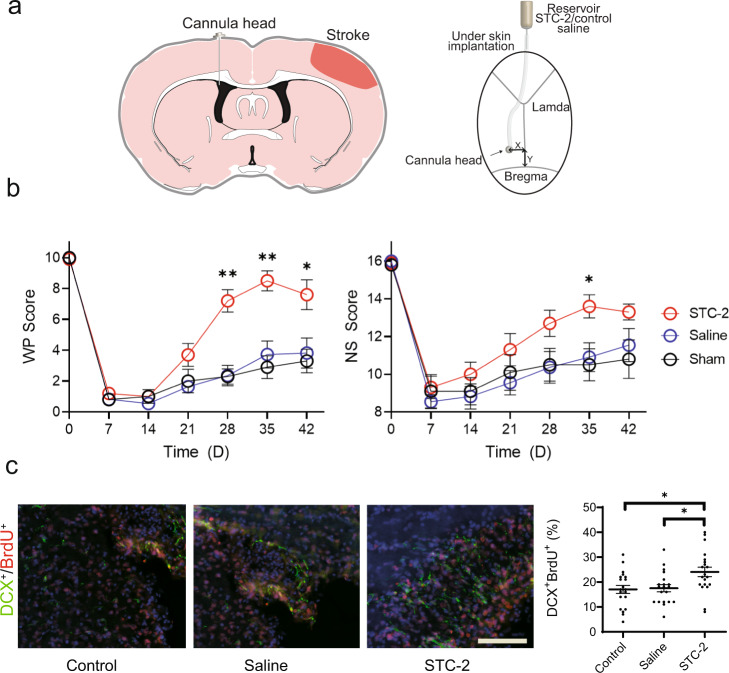
Intraventricular delivery of recombinant STC2 protein in rodents. **a** Schematic of the brain slice view (left) and top view (right) in the skull for STC2 protein delivery via mini-osmotic pump. The cannula of the osmotic pump is implanted at 0.8 mm posterior (Y) and 1.5 mm contralateral (X) to the bregma at a depth of 3.5 mm at 1-week after stroke. **b** Behavioral testing. Vibrissae-forepaw (WP) behavioral testing (left) and overall neurological score (right). Based on WP, STC2 group exhibited statistically significantly greater recovery beginning at 4 weeks post-stroke compared to other groups. **c** Endogenous neuroblasts. Representative images of DCX + /BrdU+ cells (left) and the cell count of number co-positive cells (right) at peri-infarct region. **b** Analyzed using a log-transform two-way repeated measures ANOVA which revealed a statistically significant interaction between the effects of treatment group and WP Score (*F* [12, 162] = 7.058, *P* < 0.0001); followed by Dunnett’s multiple comparisons test. Neurological scores (NS) were analyzed using a Kruskal–Wallis test (*P* = 0.018), followed by Dunn’s multiple comparisons test. For both, *P < 0.05, **P < 0.01, data shown as mean ± SEM, *n* = 10 per group. **c** Analyzed using one-way ANOVA, followed by Tukey’s HSD post-hoc test with *P < 0.05. Data shown as mean ± SEM, *n* = 20 images/4 rats.

To further investigate the effect of STC2 alone on stroke recovery, we intraventricularly administered recombinant STC2 1-week post-stroke (Fig. 5). Intraventricular administration facilitates circulation to various regions of brain such as the SVZ and peri-infarct area via the cerebrospinal fluid^40,41^ (Supplementary Fig. 18). We found that administration of STC2 leads to an improvement in functional recovery (Fig. 5b, Supplementary Figs. 19 and 20) and to an increase in endogenous neuroblasts migrating toward the peri-infarct region (BrdU, with Doublecortin at 6 weeks post-stroke, Fig. 5c). No change in the quantity of less mature mitotic markers (BrdU, with PAX6 and Nestin, Supplementary Fig. 21) were observed at the 6-week timepoint. These experiments demonstrate that although STC2 may be an essential pathway constituent that augments improved functional recovery, further experiments are needed to establish a direct causal link between STC2 and endogenous stem cell proliferation in vivo.

## Discussion

Here we introduce a conductive polymer system that can manipulate the neural environment to improve recovery after stroke. We show that electrical modulation of NPC transplants improves functional recovery following stroke more than either electrical modulation or NPC transplant therapy alone. This suggests that optimizing multiple factors of the recovering stroke environment is more robust than targeting a single pathway. Moreover, the polymeric nature of the system creates the ability to intimately interact with the transplanted NPCs as well as the post-stroke tissue. Our findings demonstrate that NPCs can be optimized and made more effective as treatments for neural recovery.

In vivo electrical stimulation of transplanted neural stem cells alters the repair mechanisms after stroke. We found that electrical stimulation alters the transcriptome of NPCs, and that STC2 is a critical upregulated molecule. STC2 is a secreted, homodimeric glycoprotein that is expressed in a wide variety of tissues including neurons with autocrine or paracrine functions^36,37,42,43^. STC2 is primarily known for its role in cell turnover, specifically in tumors^44–46^, and has been demonstrated to confer neuroprotective effects^37–39,46–48^. Lentiviral transduction of NPCs with STC2 gain and loss of function demonstrates that STC2 is a critical mediator for enhanced functional recovery after stroke (Fig. 4). Moreover, direct administration of recombinant STC2 leads to an increase in migratory neuroblasts toward the penumbral region and an improvement in behavioral outcome in our rodent stroke model. Further mechanistic studies are needed to determine a causal mechanism, and more specifically, to investigate how STC2 production by the electrically stimulated NPCs results in increased endogenous stem cell production.

While brain plasticity declines over time, intrinsic repair mechanisms such as endogenous stem cell production remain and are currently being explored for therapeutic applications^8,11,28,47^ Utilizing platforms such as the conductive polymer system described here provides an opportunity to increase this intrinsic response at critical time points during recovery to improve the brain’s ability to heal after injury. Electrical modulation of transplanted stem cells greatly improves sensorimotor stroke recovery in young adult rats (Fig. 1c, d). Although we observed minimal NPC survival in the neural environment at 6 weeks post-stroke (Supplementary Fig. 7), we suggest that their initial subsistence throughout stimulation is of sufficient duration to transiently release trophic factors that may influence endogenous NSCs. The increased endogenous stem cell production observed in these animals suggests one possible mechanism for this trophic factor-mediated improvement in recovery.

Given that there are major differences in adult neurogenesis between humans and rodents^48^, it is important to acknowledge that although neurogenesis persists into adulthood in various regions of the human brain^49,50^, the physiological significance of newly generated neurons as well as the extent and the implication of neurogenesis in humans remains to be fully elucidated. Another limitation factor is that the majority of strokes occur in the aged population. Studies have shown that aged animals recover differently from stroke^51^. The number of endogenous NPCs produced also decreases over time^52–54^ Further examination of stroke recovery and endogenous stem cell production in aged animals will help advance these findings towards clinical applications. Lastly, this study was focused on investigating the effect of electrical stimulation when the transplanted stem cells were most viable (the first three days after transplantation). Stroke repair mechanisms via chronic stimulation of brain tissues were not investigated here and is a possible area warranting further exploration with this conductive polymer system.

The primary premise of this work is that a conductive polymer system used to modulate transplanted NPCs creates an innovative method to shape the healing brain. By understanding how this modulation alters NPCs in the post-stroke brain, advances can be made in understanding how the brain recovers from stroke. Moreover, the interactive polymeric system creates the ability to identify important repair pathways that can potentially be developed independently for new stroke therapies such as STC2. Taken together, this system enables the development of new types of treatments that incorporate exogenous stimulation and endogenous repair pathways to restore function in the post-stroke environment.

## Methods

### Fabrication of the in vivo conductive scaffold system

PPy (Sigma-Aldrich) was electroplated onto indium tin oxide (ITO) slides (Delta Technologies) as described previously^22,25^. Briefly, a current density of 1 mA/cm^2^ was applied between the ITO template and a platinum reference electrode in a 0.2 M PPy and 0.2 M NaDBS (Sigma-Aldrich) electrodeposition solution at 25 °C. After removal from the ITO, the conductive scaffold was clamped between pieces of polydimethylsiloxane (PDMS; Sylgard, Dow) with a chamber slide forming cell chambers (Lab-Tek, Thermo Fisher) as previously detailed^55^. Wires were attached to the conductive scaffold outside of the media-containing chambers. For implantation, the cell chambers and PDMS were unclamped and separated from the conductive scaffold. The dimensions of the implanted scaffolds were 1 × 3 × 0.25 mm.

### In vitro hNPC electrical stimulation

The Stem Cell Research Oversight committee at Stanford approved all stem cell procedures. Human neural progenitor cells (hNPCs, hNP1) were obtained from ArunA Biomedical and originally derived from a neuroepithelial cell lineage of WA09 human embryonic stem. hNPCs, passages 6-9, were used in these experiments and kept in AB2^TM^ Basal Neural Medium supplemented with ANS^TM^ Neural supplement (Aruna Biomedical), bFGF (50 µg/ml, Croning), L-Glutamine (200 mM, Invitrogen), and LIF (10 µg/ml, Millipore). hNPCs were plated onto the PPy scaffold on Day 1 (125,000 cells/cm^2^). For in vitro experiments including RNAseq and qPCR analysis, media was changed for both electrically stimulated and non-stimulated cell groups on Day 2. Electrically stimulated cells received electrical stimulation for 1 h on Day 2 at a variety of voltages and frequencies. The current was delivered through the PPy scaffold with wires attached to either side of the PPy scaffold outside of the cell chamber. On Day 3, cells and supernatants were collected for analysis.

For STC2 gain and loss of function, hNPCs were treated with STC2 lentiviral activation particles (sc-405292-LAC) and STC2 shRNA (h) particles (sc-44127-V) respectively as per the manufacturer protocol (Santa Cruz Biotechnology Inc., Lentiviral activation particles transduction). Control shRNA lentiviral particles (sc-108080) were the control and referred to as Scramble^KD^ in this work. The Scramble^KD^ and STC2^KD^ groups were electrically stimulated as described in the above paragraph.

### Cell viability assessments

In vitro immunostaining was performed on Day 3 (1 day after electrical stimulation applied). Cell survival was determined by a Live/Dead kit (Life Technologies). The samples were incubated with 2 µL/mL of ethidium homodimer-1 and calcein AM for about 15 mins at 37 °C in the dark. After incubation, the cells were rinsed with 1X PBS, and imaged using a fluorescent microscope (Keyence BZ-X700). Four random, representative 0.34 mm × 0.45 mm areas were analyzed, and alive and dead cells on the conductive scaffold were counted by a blinded individual with results averaged across the four areas (live cells/total cells).

Alternatively, cell viability testing using Alamar Blue has been applied to see the impact of electrical stimulation on the cells’ survival^56^. Briefly, 10% Alamar Blue cell viability reagent (Thermo Fisher Scientific) was added to each sample and incubated at 37 °C for 3 h in the dark. The experimental groups were hNPCs with or without stimulation and control. After 3 hours of incubation, the absorbance of about 100 µL per sample was measured in duplicates at 570 and 600 nm using a multi-plate reader (SpectraMax, Molecular Devices). The percentage reduction in absorbance (percentage viability) was calculated with respect to total cells as per the manufacturer protocol.

### RNA-Seq

In vitro electrically stimulated and unstimulated hNPC cDNA was isolated 24 h following electrical stimulation as described in the “in vitro hNPC electrical stimulation” section (*n* = 4 per group). RNA was extracted with the RNeasy Mini Plus kit (Qiagen, Hilden, Germany) after homogenization in Trizol (Life Technologies). cDNA was then synthesized using iScript cDNA.

Synthesis Kit (Bio-Rad, Hercules, CA),and purity was verified by the Agilent BioAnalyzer system.

Samples were prepped with Illumina TruSeq Stranded mRNA kit as per manufacturer’s suggestion and sequenced as reverse paired-end on the HiSeq-4000 sequencer conducted at the Stanford SFGF as described previously^57^. Raw fastq files was trimmed with Trimmomatic/0.36^58^ and reads were aligned to the hg38 reference genome with STAR/2.5.1b aligner^59^. Gene level counts were determined with STAR –*quantMode* option using gene annotations from GENCODE (p5)^60^. QC assessments such as unique alignment counts, unique/multiple ratio or exon/intron ratio was derived with ngsutilsj-0.3-2180ca6 using the *bam-stats* option^61^. Differential gene expression and all other pathway analysis are conducted with R/3.5.3. Samples were imported, normalized with trimmed mean of *M*-values (TMM) from the EdgeR/ 3.24.3 package^62^ and further transformed with VOOM from the Limma/ 3.38.3 package^63^. A linear model using the empirical Bayes analysis pipeline also from limma was then used to obtain *p*-values, adjusted *p*-values and log-fold changes (LogFC). Differential expressed genes (DEG) are defined with cutoffs FDR < 0.05; *p* value < 0.05 and logFC > abs (1.5).

Gene Set Enrichment Analysis (GSEA) was performed on preranked logFC using the R package fgsea/ 1.13.5^34^. Predefined pathways such as Hallmark gene sets and KEGG pathways were downloaded directly from the Molecular Signatures Database (MSigDB)^64,65^ Upon review of unsupervised clustering data using Tukey’s outlier method, one of the unstimulated samples deviated remarkably from the other samples in the same experimental cohort and was removed from further analysis.

**Table:** 

Resource/software	Source	Indentifier
Hg38 human index and gtf	GENCODE	https://www.gencodegenes.org/human/
STAR aligner^59^	–	https://github.com/alexdobin/STAR
Trimmomatic/0.36^58^	–	https://github.com/timflutre/trimmomatic
ngsutilsj-0.3-2180ca6	–	https://github.com/compgen-io/ngsutilsj
EdgeR^60^	Bioconductor	https://bioconductor.org/packages/release/bioc/html/edgeR.html
Limma^62^	Bioconductor	http://bioconductor.org/packages/release/bioc/html/limma.html
fgsea^66^	Bioconductor	https://bioconductor.org/packages/release/bioc/html/fgsea.html
MSigDB^64^	Broad Institute	http://software.broadinstitute.org/gsea/msigdb

### RNA extraction and qPCR

Total RNA was extracted from cells using a Qiagen RNeasy Plus Micro Kit (Qiagen). After accomplishing first-strand cDNA synthesis by iScript cDNA Synthesis Kit (Bio-Rad, Hercules, CA), quantitative real-time polymerase chain reaction (qRT-PCR) was performed with Taqman-polymerase and primers (Qiagen) for gene expression analysis. qRT-PCR was carried out on a QuantStduio 6 Flex Real-Time PCR System (ThermoFisher, Waltham, MA). The Delta-Delta CT method was utilized for relative expression levels with GAPDH as a housekeeping gene Taq polymerase and Taqman primers (Life Technologies) for GAPDH (Hs), STC2 (Hs01063215_m1), SNCB (Hs00608185_m1), NRN1 (Hs00213192_m1), TNNT1 (Hs00162848_m1), PLOD2 (Hs01118190_m1), PDEC4C (Hs00971865_m1), PPF1A4 (Hs00949811_m1), TMEM45A (Hs01046616_m1), and FGF11 (Hs00182803_m1) formed the qPCR reaction mixtures. The Delta-Delta CT method was utilized for qPCR analysis with the *TUBB* housekeeping gene and hNPCs grown on a glass chamber slide for 3 days as references.

### ELISA STC2 and In-cell western blot analysis

Supernatant was collected from the hNPCs in vitro, on Day 3 after plating. A human STC2 ELISA kit (AB222880, Abcam) was used to assess STC2 concentrations according to manufacturer instructions. Samples were performed in duplicate with *n* = 4 for each group.

For the in-cell western assay, the subcellular level of STC2 was quantified in situ using infrared (IR) intensity. After the electrical stimulation of STC2, the cells were plated in a 96-well plate (20,000 cells/well) and were immunolabeled with an IR-conjugated secondary antibody (IRDye® Secondary Antibodies, LiCor) using the standard immunocytofluorescence protocol. After the completion of the staining procedure, the plate was imaged using an Odyssey Fc IR imaging system (LiCor, Lincoln, NE). STC2 intensity was normalized to GAPDH expression by using the Odyssey CLx Imaging Studio 3.1 Analysis software.

### dMCA occlusion and cell implantation

All animal procedures were approved by Stanford University’s Administrative Panel on Laboratory Animal Care. Adult, male T-cell deficient nude rats (NIH-RNU 230 ± 30 g)^67^ underwent distal middle cerebral artery (dMCA) occlusion model with occlusion of both common carotid arteries lasting 30 min as described previously^68^. Rats were anesthetized with isoflurane with buprenorphine administered subcutaneously for analgesia. Ampicillin was in cage water 1 day prior to surgery (1 mg/ml) and for 7 days after transplantation.

One week after stroke, animals were randomized by vibrissae-whisker paw score, and implantation surgeries performed by a blinded individual. A craniectomy was drilled above the left cortical region between the neuroanatomical lambda and bregma markers, and the dural layer was excised. The conductive polymer system (cultured with hNPC cells for 24 h; without any in vitro electrical stimulation) was removed from the in vitro system and implanted over the exposed brain tissue primarily on the penumbral cortex medial to the lesion after a phosphate buffered saline (PBS) wash (with ~5 × 10^4^ cells in hNPC groups or media alone for other groups). Surgicel (Ethicon) was placed over the implant to prevent movement with skin closure. A reference electrode attached to the cannula system was placed on the contralateral skull (Fig. 1). The main cannula was secured to the contralateral skull with dental cement. Sham groups included animals with the dura opened but no implant.

For STC2 intraventricular delivery experiments, adult Sprague Dawley rats (300 ± 30 g) underwent dMCA occlusion as described in the above paragraph, followed by STC2 osmotic pump implantation. About 24 h prior to implantation, recombinant human STC2 protein (4 ng/mL of 1X PBS, Novus Biologicals) was loaded on to the osmotic pump (Azlet mini-osmotic pump model 2001, Braintree Scientific) and incubated at 37 C as per the manufacturer protocol. The cannula from the osmotic pump was implanted at 0.8 mm posterior, 1.5 mm contralateral, and 3.5 mm depth with respect to bregma. The reservoir was stored under the skin near the neck (back side). Control groups included a Sham (that underwent stroke, no treatment), and Saline (injected with 1X PBS without any STC2 protein in the osmotic pump).

### In vivo hNPC electrical stimulation

Electrical stimulation (±800 mV, 100 Hz for 1 h) was applied to the rats receiving electrical stimulation for the first 3 days after implantation (implantation on Day 8, electrical stimulation on Day 9, Day10, and Day 11 post-implantation). The potential was applied across the polymer and the reference electrode while the animals were anesthetized as described above using a function generator (E3641A, Agilent).

### Stroke volume and slice immunohistochemistry

At 3- or 6-week post-stroke, rats were perfused 4% paraformaldehyde (Sigma Aldrich) and 40 µm coronal slices were sectioned as described previously^68^. Animals perfused at the 6-week time point were used for stroke volume analysis. Animals perfused at 3 weeks post-stroke were used for BrdU and immunostaining analysis.

BrdU intraperitoneal injections were performed 24 h after the implantation of the conductive polymer system (post-stroke day 9). Briefly, BrdU was diluted in PBS to make a sterile solution of 10 mg/mL. A concentration of 100 mg/kg was intraperitoneally injected. Animals were killed and fixed at 3 weeks post-stroke. Primary antibodies were incubated in blocking buffer at 4 °C overnight, followed by three 15-min PBS washes and detected by secondary antibodies (Alexa Fluor 488, 555, or 647, Thermofisher Scientific) as described previously^68^. Samples were counter-stained with DAPI (Sigma-Aldrich) to visualize nuclei and mounted with Fluoromount Aqueous Medium (Sigma-Aldrich) before imaging. Samples were imaged on a Keyence All-in-One Fluorescence Microscope (BZ-X700, Keyence) using ×20 or ×60 objectives. Primary antibodies include anti-BrdU (1:200, Abcam), anti-GFAP (1:1000, Millipore Sigma), anti-Pax6 (1:100, Fisher Scientific), anti-NeuN (1:500, Cell Signaling Technology), anti-Nestin (1:200, Millipore Sigma), anti-PECAM (1:100, Millipore Sigma), and anti-TUJ1 (1:100, Neuromics). Secondary antibodies were added as noted above. Images were analyzed on a Keyence optical microscope.

To assess for BrdU stained cells in the rat tissue, serial slices were taken 400 µm apart from the genu of the corpus collosum to the splenium. Cell counting were performed by a blinded observer. Peri-infarct area was defined as the area immediately surrounding the infarcted tissue and sampled in a 750 µm × 650 µm window. The SVZ region was defined in the same manner as previously^69^, briefly tissue next to the ventricles with higher cellular density was assessed and 750 µm x 650 µm frame was obtained using a Keyence All-in-One Fluorescence Microscope (BZ-X700, Keyence) using ×20 objectives. The midpoint between the peri-infarct region and SVZ region was then defined that as the middle area and assessed in the same manner. The threshold was then selected on one representative image and the same parameters were set for all images for counting purposes. Background signal was ignored, and the cells representative of true signal were then stereologically counted and averaged across slices for a given animal with *n* = 4 per experimental group.

Stroke volume was assessed using cresyl violet staining 5 weeks after stroke as described previously^68^. Serial slices were taken 400 µm apart from the genu of the corpus collosum to the splenium. Areas were calculated using the following equation $\frac{{Area}\;{Contalateral\,-\,Areal}\;{Psilateral}}{{Area}\;{Contralateral}}\times 100.\;$Assessments were performed by a blinded individual.

For quantification of TUJ1 (1:100, Neuromics) and GFAP (1:00, Invitrogen), four representative peri-infarct areas were selected from each ipsilateral slice at 400 µm intervals. These were selected at the same point in each slice by a blinded individual. ImageJ software was used by a blinded individual to calculate average fluorescence intensity against Tuj1 and GFAP.

### Behavior analysis

Animals were divided into matched groups based on pre-transplant behavior testing (*n* = 10 per group) and behavior testing was performed by blinded individuals. Functional recovery was assessed in two ways: (1) Using the modified neurologic severity scale (NSS)^27,70^ (Supplementary Table 4, a composite score assessing motor and sensory function including normal walking, walking on a beam, tactile and visual response when paw muscles are stimulated by touch) and (2) vibrissae-forepaw test^71^. Animals were trained on three separate days prior to recording their baseline behavior. After baseline, the animals underwent dMCA occlusion and were tested 1 week after stroke prior to implantation. For the STC2 experiments, two animals died during the follow up and were removed from analysis (1 in the Sham and one in the STC2^KD^). Animals without a significant deficit (significant deficit = vibrissae-forepaw score prior to implantation at <30% of baseline) were removed. The vibrissae-forepaw behavioral assay was evaluated for the potential confound of testing effect (by repeated measurements) via longitudinal assessments of the limb ipsilateral to the stroke as a non-effected control (Supplementary Fig. 22). The results do not suggest the presence of a testing effect however we acknowledge the limitation of this control being performed in stroked animals.

### Statistical analysis

Statistical analysis was performed in GraphPad Prism V6.1+ or with genomic software. For comparisons of means in samples with normal distributions and homogeneous variances (as indicated by a Levene’s test), an unpaired *t* test was used for two groups. For more than two groups an ANOVA was used for comparisons between means (or two-way repeated measures ANOVA, where appropriate), followed by Tukey’s HSD (or Dunnett’s, where appropriate) for multiple comparisons. In cases of a non-normal distribution (as indicated by a Shapiro–Wilk test) or unequal variances (Levene’s test), a nonparametric Mann–Whitney test (with Benjamini-Hochberg correction to control the false discovery rate at the 0.05 level, in incidences of repeated measure comparison) or a Welch’s *t* test was used for comparisons between two means, and a nonparametric Kruskal–Wallis test was used for comparisons between more than two means, followed by Dunn’s test for multiple comparisons. For RNA-Seq pathway analysis, a false discovery analysis was used to correct for multiple variables. Data are presented as mean ± SEM. Significance was ascribed at *p* < 0.05.

### Reporting summary

Further information on research design is available in the Nature Research Reporting Summary linked to this article.

## Supplementary information

Supplementary Information

Reporting Summary

## Data Availability

Source data are provided with this paper. RNA sequencing data has been posted to the Gene Expression Omnibus (GEO Accession #GSE185402) [Accession]. Additional data is available from the corresponding author upon request. Source data are provided with this paper.

## Source data

Source Data
