# Supplementary material for: Electrical modulation of transplanted stem cells improves functional recovery in a rodent model of stroke
Source: Nat Commun. 2022 Mar 15;13:1366. doi: 10.1038/s41467-022-29017-w (PMC8924243; doi:10.1038/s41467-022-29017-w)
Supplement: Supplementary file 1 — Supplementary Information [file 41467_2022_29017_MOESM1_ESM.pdf]

Supplementary Notes

Electrical Stimulation Optimization

Previous *in vitro* stimulation of NPCs showed changes in VEGFA [1]. Using VEGFA as a gene of interest, we electrically stimulated the cells across various voltages (±200mV, 400mV, or 800mV square wave) and frequencies (100 and 200Hz) and monitored the response of VEGFA (Supplementary Fig. 2a). Given that the largest change in VEGFA expression was observed with electrical stimulation of ±800mV at 100Hz, we used these stimulation parameters for our *in vitro* and *in vivo* stimulation paradigms. Under these parameters, cell viability was assessed with Alamar Blue and Live/Dead staining (Supplementary Fig. 2b-d). These viability assays demonstrated that cell viability did not significantly change with electrical stimulation.

Stroke Size Evaluation

The stroke size was evaluated at 6 weeks post-stroke. Given that interventions were performed 1-week post-stroke, any potential neuroprotective effects that reduce stroke size were highly unlikely. Consistent with this, no significant difference in stroke sizes were observed between any of the groups in our experiments (Supplementary Figs. 6 and 17).

Stroke and Peri-infarct Cell Analysis

To assess further influences of the combined electrical stimulation and stem cell therapies, we evaluated cell type in the peri-infarct and stroke regions. At the 6-week post-stroke time point, no change in the number of neurons or glia in the stroke or peri-infarct regions were observed (Supplementary Fig. 10).

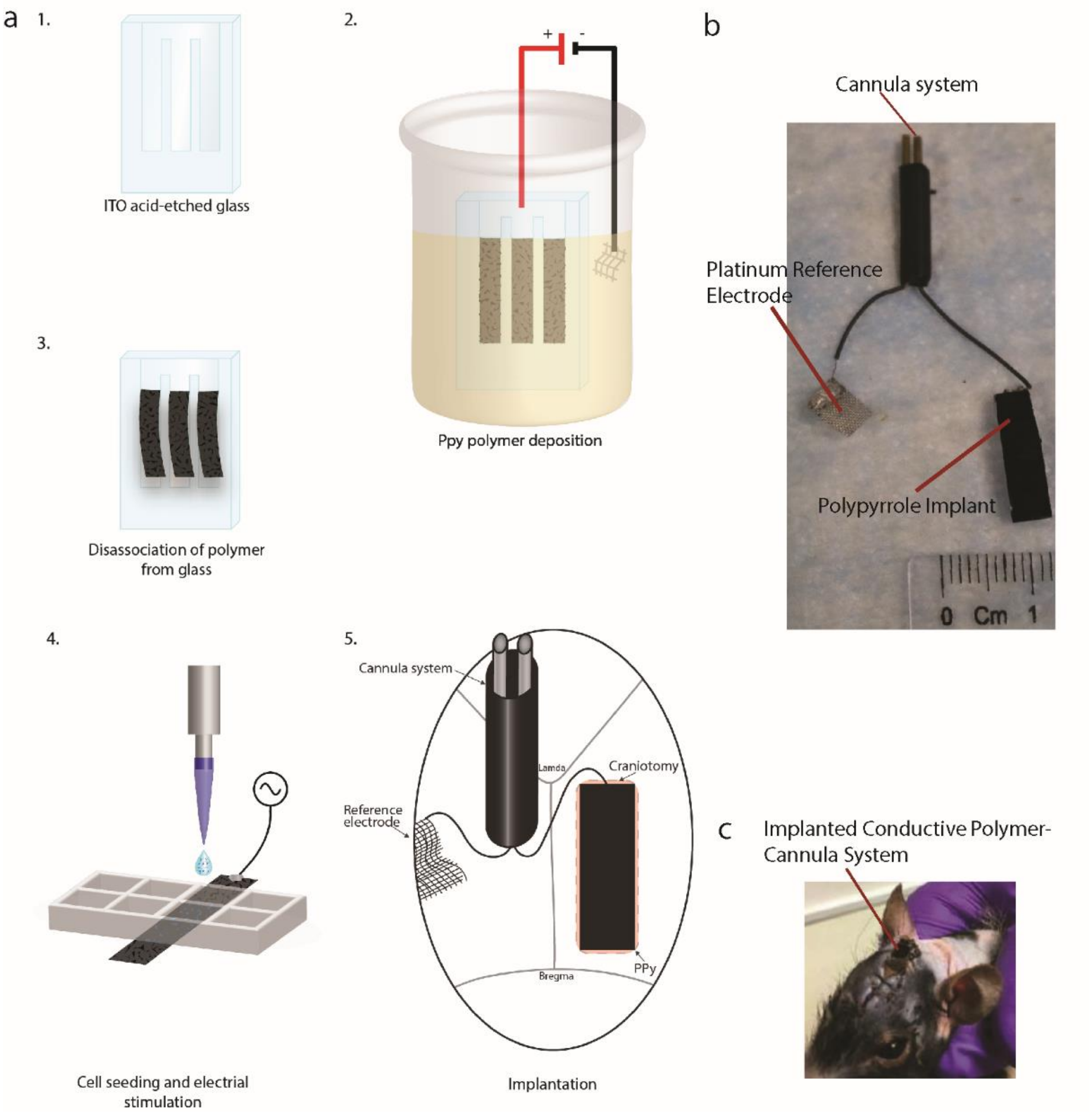

Supplementary Fig. 1 Conductive-Polymer stem cell system. a, Schematic fabrication of implantable conductive polymer-stem cell system (PPY – polypyrrole scaffold). **b,** Photograph of cannula system. **c,** Photograph of animal with cannula in place.

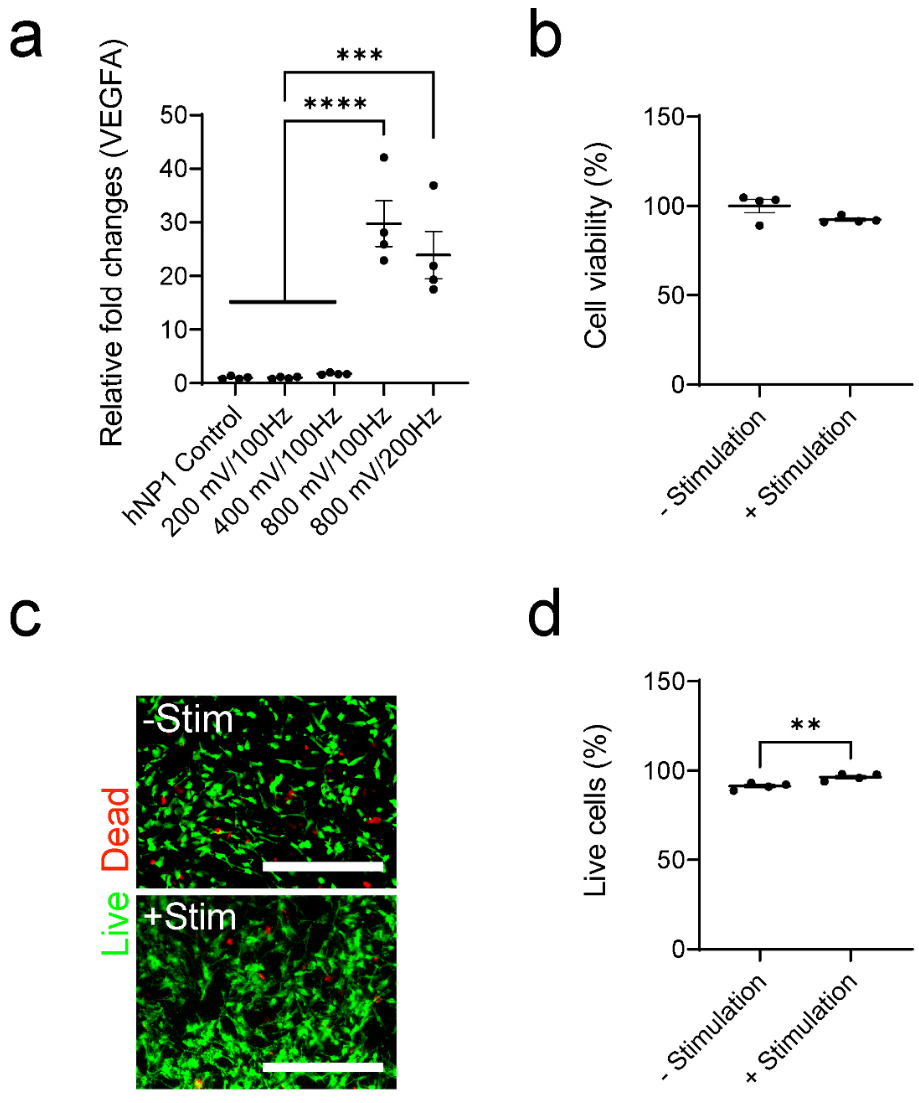

Supplementary Fig. 2 Optimization of electrical stimulation parameters on NPCs. a, NPCs expressed significantly higher VEGFA in the 800mV/100Hz and 200Hz stimulations for 1 hr. *** indicates statistical significance compared to control (P<0.001, data shown as mean±SEM, n=4). **b,** Cell metabolic viability assay measured via Alamar Blue showing no difference in cell survival between stimulated and unstimulated cells at 800mV with 100 Hz frequency for 1 hr (data shown as mean±SEM, n=4). **c,** Representative images of fluorescently-labeled cells stained using a live/dead assay kit. Green (calcein AM) and red (ethidium homodimer-1) indicate viable and dead cells, respectively. Scale bar indicates 100 µm. **d,** Quantification of the percentage of live cells from live/dead assay (in fig. 2c) showing no significant difference in living cells after electrical stimulation (800mV at 100Hz for 1 hr, data shown as mean±SEM (n=4). **a, b, d.** Analyzed using a one-way ANOVA, followed by Tukey's HSD post hoc test with ** P<0.01, *** P<0.001 and **** P<0.0001.

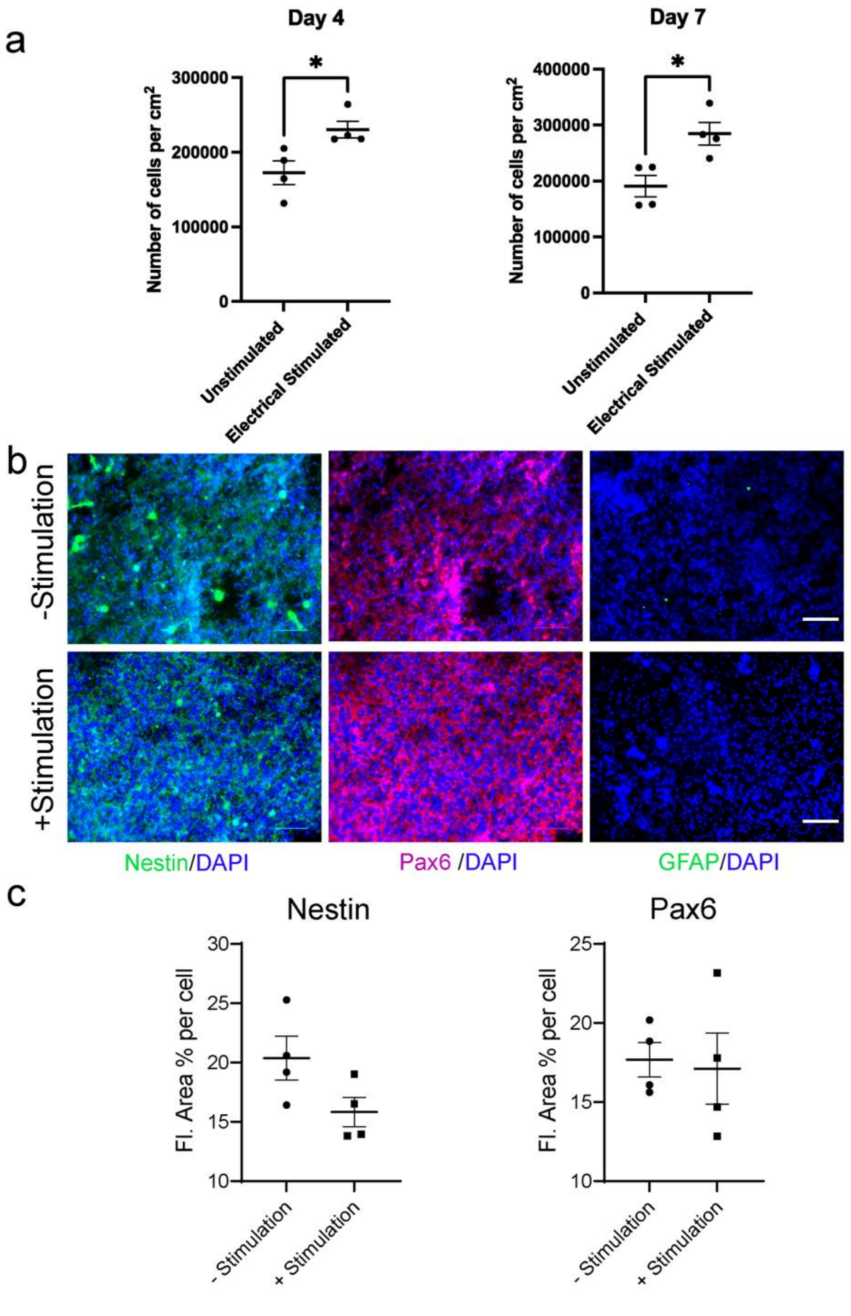

Supplementary Fig. 3 Impact of electrical stimulation on NPC biology. a, NPCs proliferated significantly when electrically stimulated compared to un-stimulated groups for at least 7-days. * indicates a statistically significant difference with p-value <0.05 when analyzed using an unpaired two-tailed t-test. Data shown as mean±SEM, n=4. **b,** Representative images of fluorescently-labeled cells stained for mature mitotic markers – Nestin and Pax6, and glial marker – GFAP. Blue represents nucleus. Scale bar indicates 100 µm. (Data shown as mean±SEM, n=4) **c,** Quantification of percentage area under fluorescence per cell from histology (in fig. 2b) showing no significant difference in Nestin and Pax6 after electrical stimulation (800mV at 100Hz for 1 hr, data shown as mean±SEM, n=4). There were no countable number of cells that stained for GFAP. Analyzed using an unpaired t-test with a two-tailed p-value > 0.05.

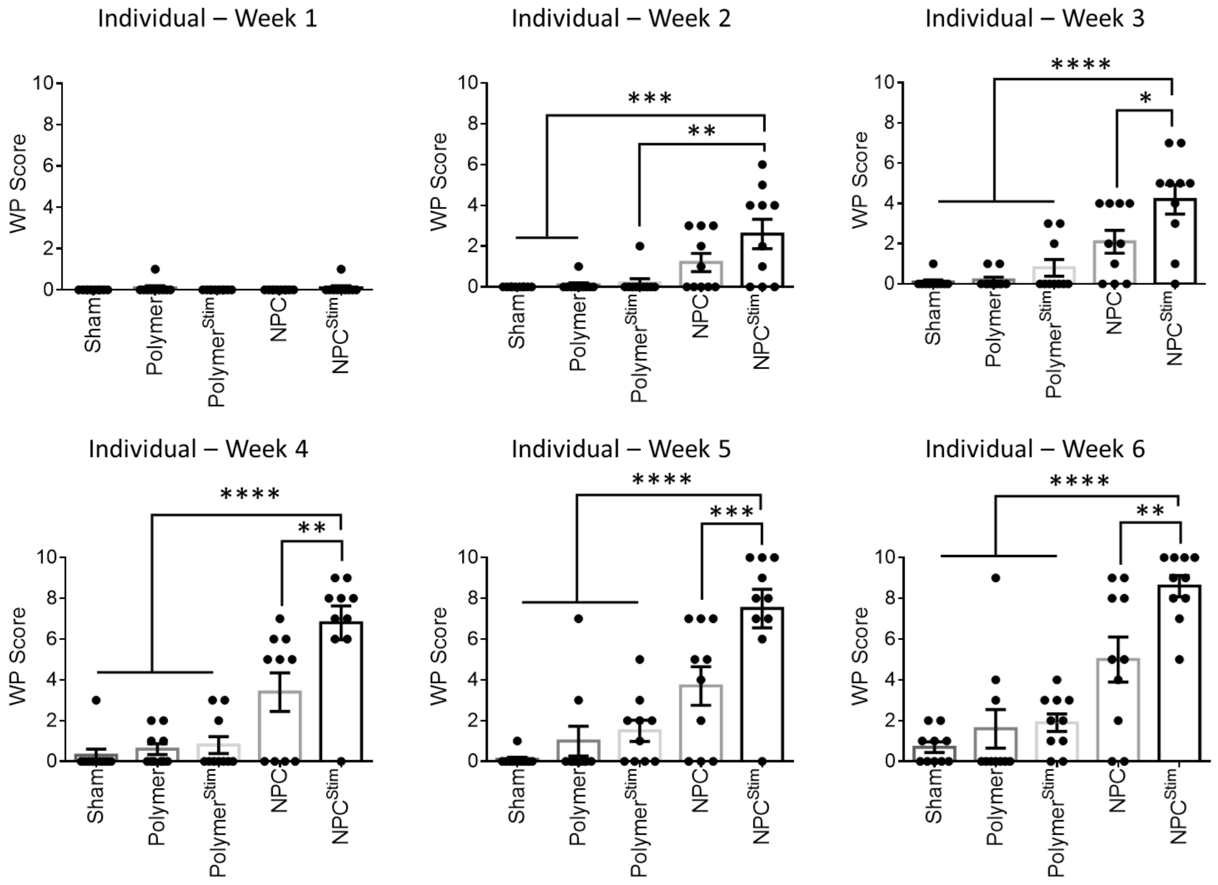

Supplementary Fig. 4 Conductive-Polymer stem cell system improves functional stroke recovery. Vibrissae-forepaw (WP) behavioral testing for individual weeks. NPC^{Stim} group exhibits improved recovery starting at as early as 1-week post stimulation (2-weeks post-stroke) compared to control. Analyzed using log-transform two-way repeated measures ANOVA followed by Dunnett's multiple comparisons test, * $p< 0.05$, ** $p < 0.01$, *** $p<0.001$, **** $p<0.0001$, data shown as mean±SEM, n=10 per group.

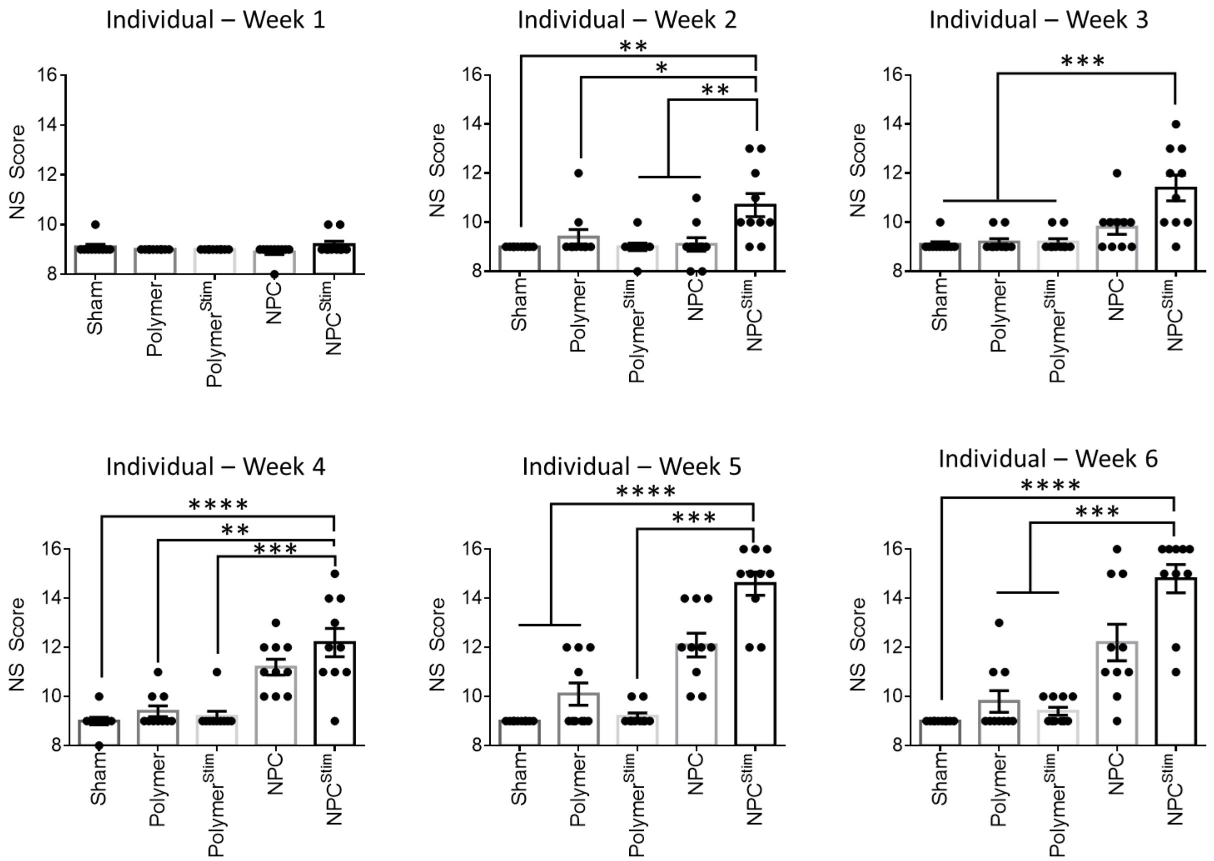

Supplementary Fig. 5 Total neurological score (NS) testing. Neurological score (NS) behavioral testing for individual weeks. NPC^{Stim} group exhibits improved recovery starting at as early as 1-week post stimulation (2-weeks post-stroke) compared to control. Analyzed using Kruskal-Wallis followed by Dunn's multiple comparisons test, * $p< 0.05$, ** $p < 0.01$, *** $p<0.001$, **** $p<0.0001$, data shown as mean±SEM, n=10 per group)

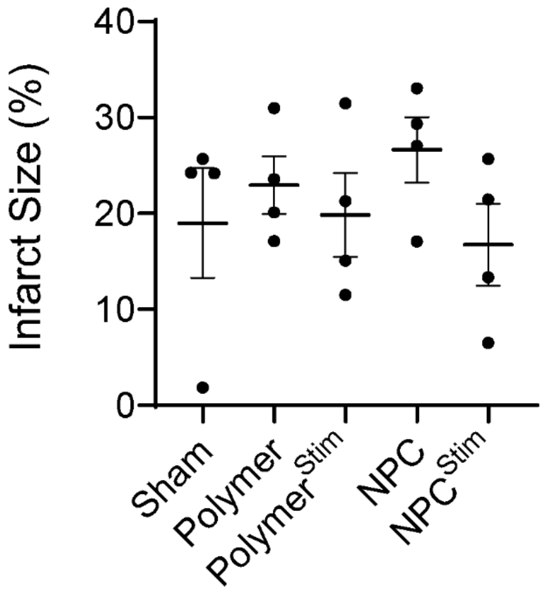

Supplementary Fig. 6 Infarct size. Percentage calculation of infarct size for Sham, Polymer, Polymer^{Stim}, NPC, and NPC^{Stim} animal groups. There were no statistically significant differences between any group. Analyzed using a one-way ANOVA, followed by Tukey's HSD post hoc test, data shown as mean±SEM, n=4 per group.

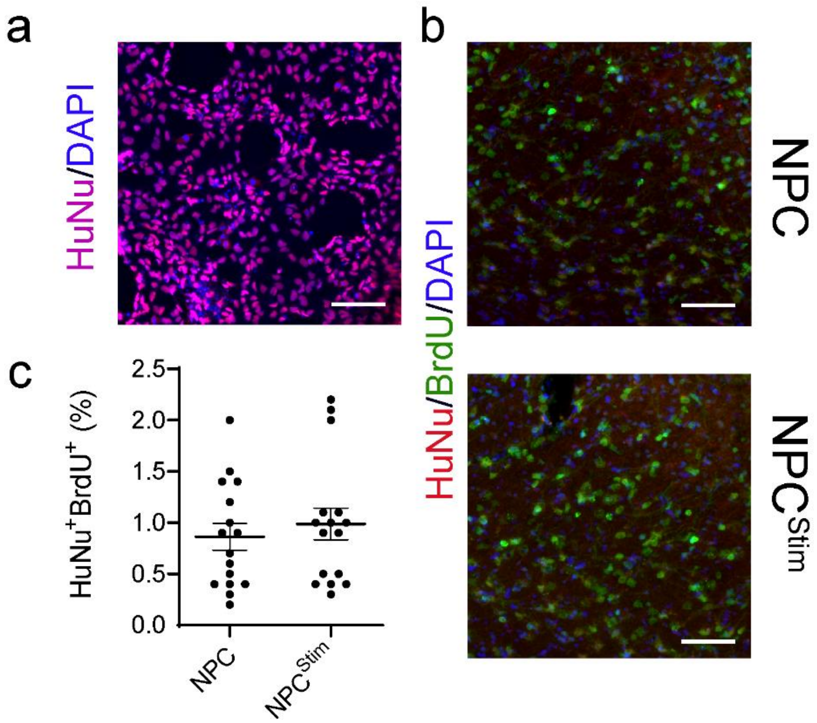

Supplementary Fig. 7 Neural stem cell characterizations. a, The human neural stem cells stain well for human nuclear stain (HuNu) *in vitro.* **b,** Representative images of brain slices stained for HuNu (red), BrdU (green) and DAPI (blue). Scale bar indicates 100 µm. **c,** Cell count analysis for $HuNu^{+}BrdU^{+}$ cells in the peri-infarct region of the brain slices showing that around 1% of $BrdU^{+}$ cells stained for $HuNu^{+}$. Analyzed using an unpaired t-test with a two-tailed p-value > 0.05, data shown as average ±SEM, n=16 images/4 rats.

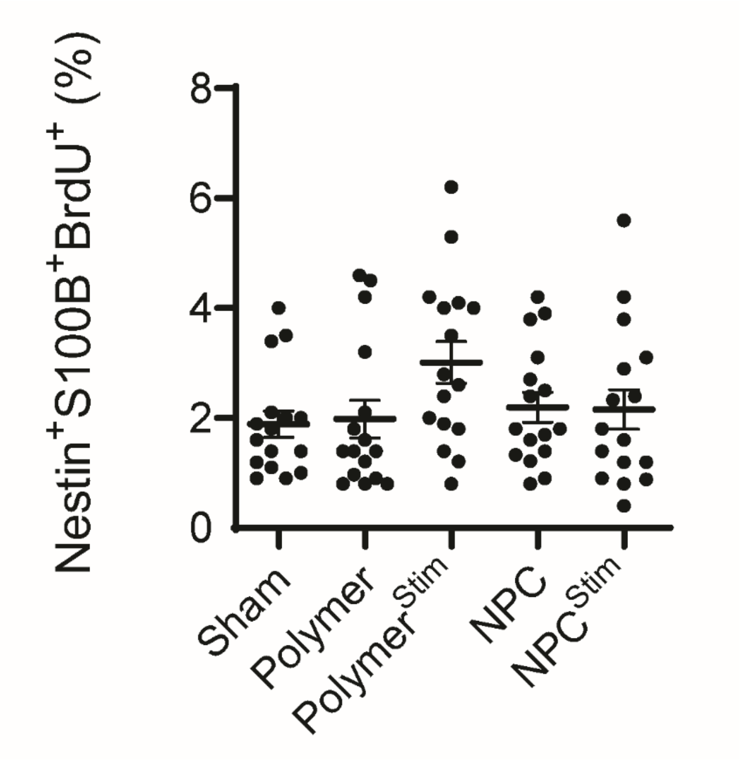

Supplementary Fig. 8 Cell count analysis of co-labeled post-stroke brain slices. Cell counts of Nestin^{+}/S100B^{+}/BrdU^{+} cells in the peri-infarct (PI) tissue. There were no statistically significant differences between any group. Analyzed using a one-way ANOVA, followed by Tukey's HSD post hoc test, data shown as average ±SEM, n=16 images/4 rats.

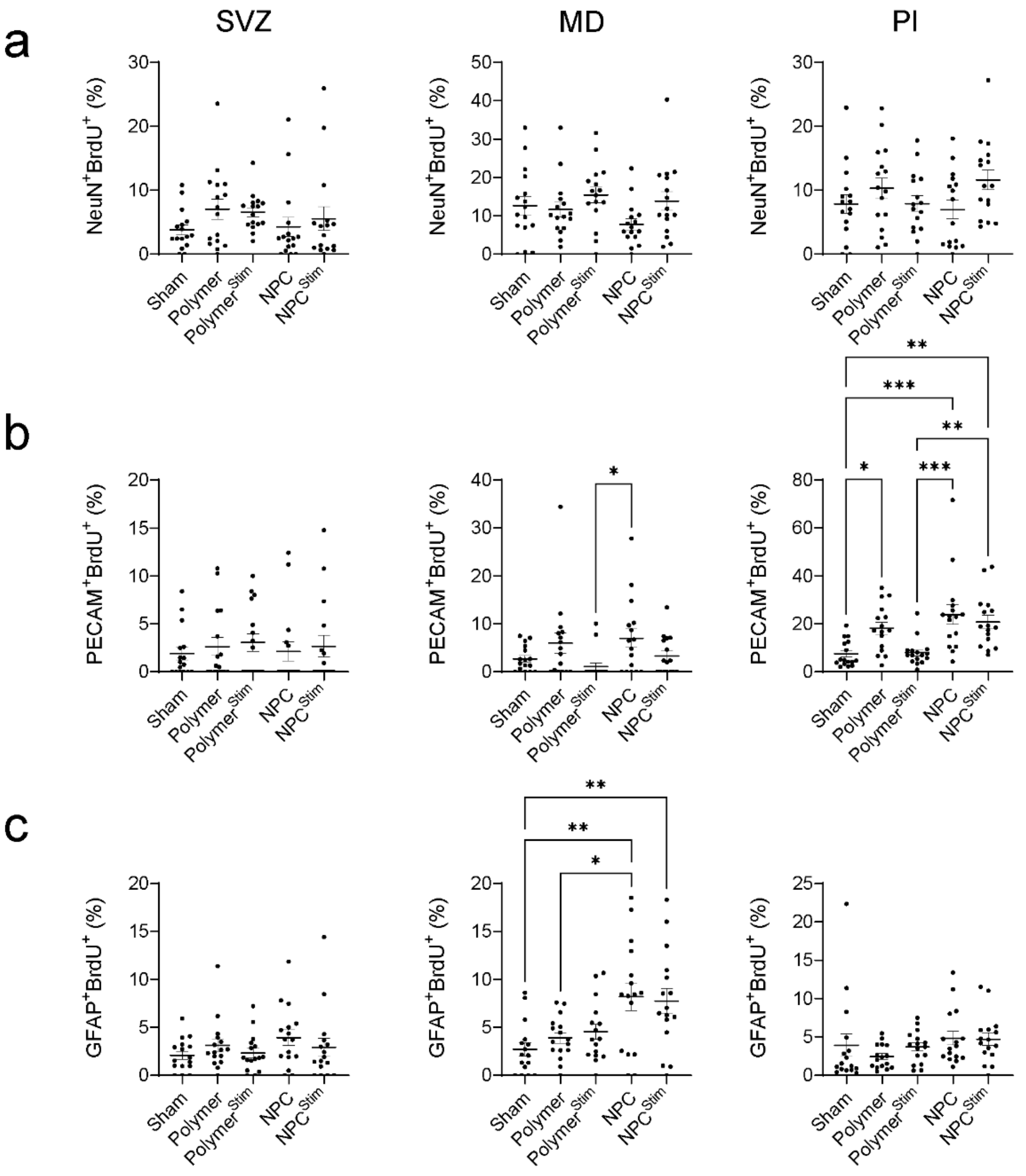

Supplementary Fig. 9 Cell count analysis of co-labeled post-stroke brain slices. a, Cell counts of NeuN$^+$/BrdU$^+$; **b,** PECAM$^+$BrdU$^+$; and **c,** GFAP$^+$/BrdU$^+$ cells in the subventricular (SVZ), middle (MD), and peri-infarct (PI) tissue. Analyzed using a one-way ANOVA, followed by Tukey's HSD post hoc test with * <0.05, ** P<0.01, and *** P<0.001, data shown as average ±SEM, n=16 images/4 rats.

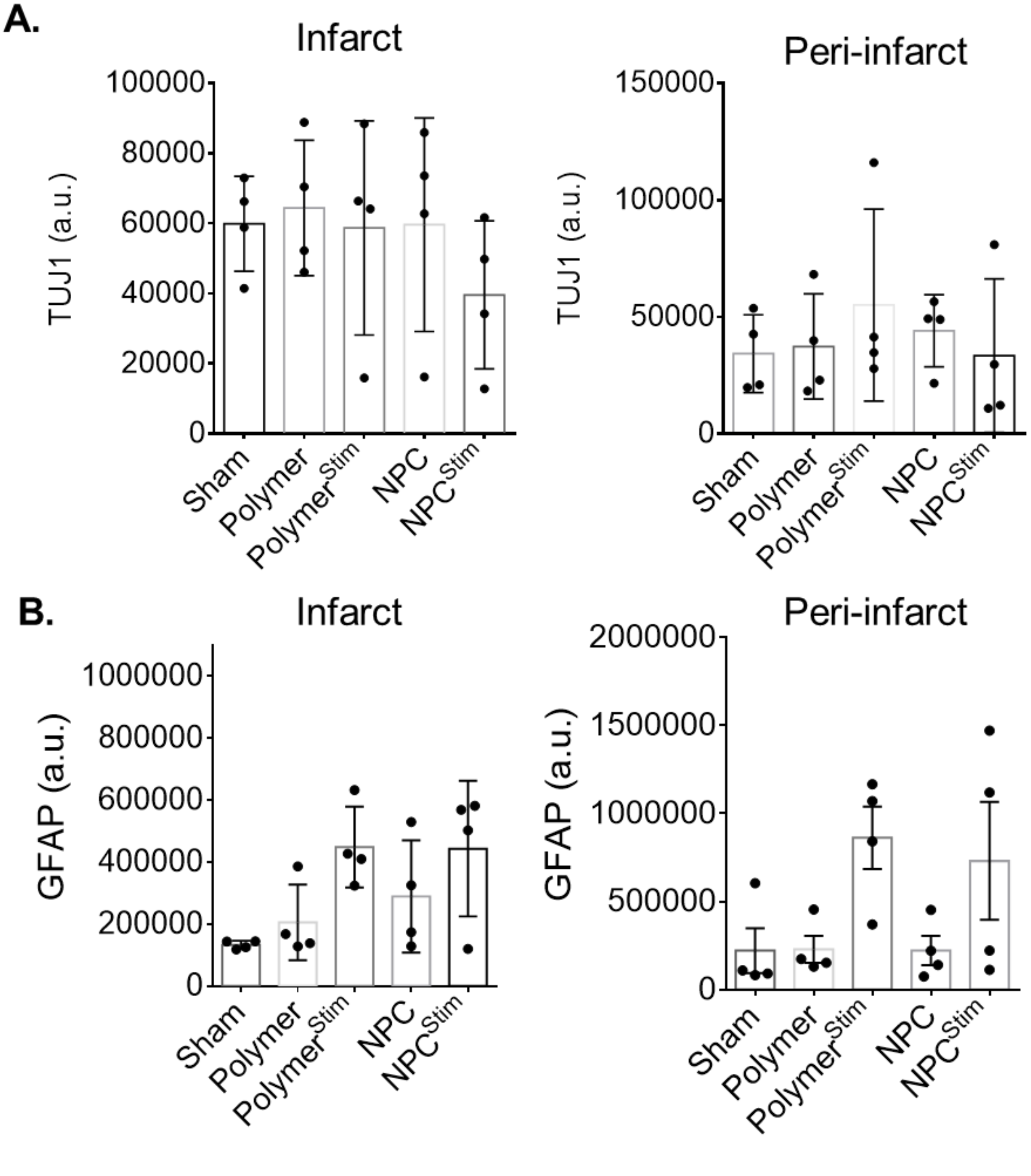

Supplementary Fig. 10 Quantification of neuronal and glial staining at 6 weeks post-stroke. Quantification of area of **a,** TUJ1 and **b,** GFAP staining in brain tissue at the infarct and peri-infarct region at the 6-week time point (a.u. = arbitrary units; data shown as mean±SEM, n=4, analyzed using a one-way ANOVA, followed by Tukey's HSD post hoc test).

a

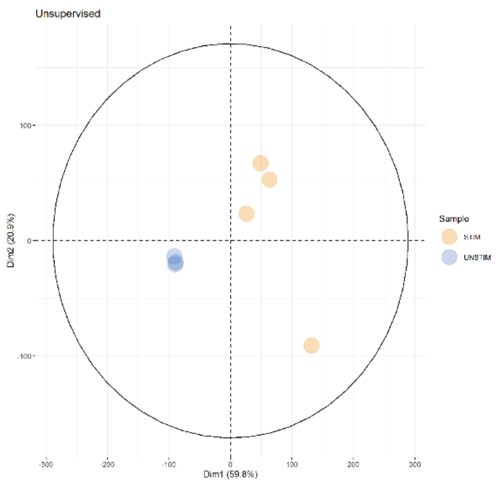

b

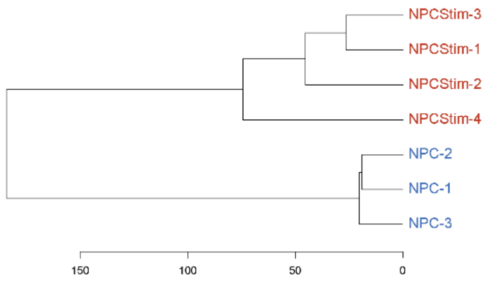

Supplementary Fig. 11 Transcriptome Analysis. a, Unsupervised principal component analysis (PCA) of unstimulated (blue, NPC) and electrically stimulated (red, NPC^{Stim}) neural progenitor cells showing separation of groups. **b,** Unsupervised hierarchical clustering of unstimulated and electrically stimulated NPCs.

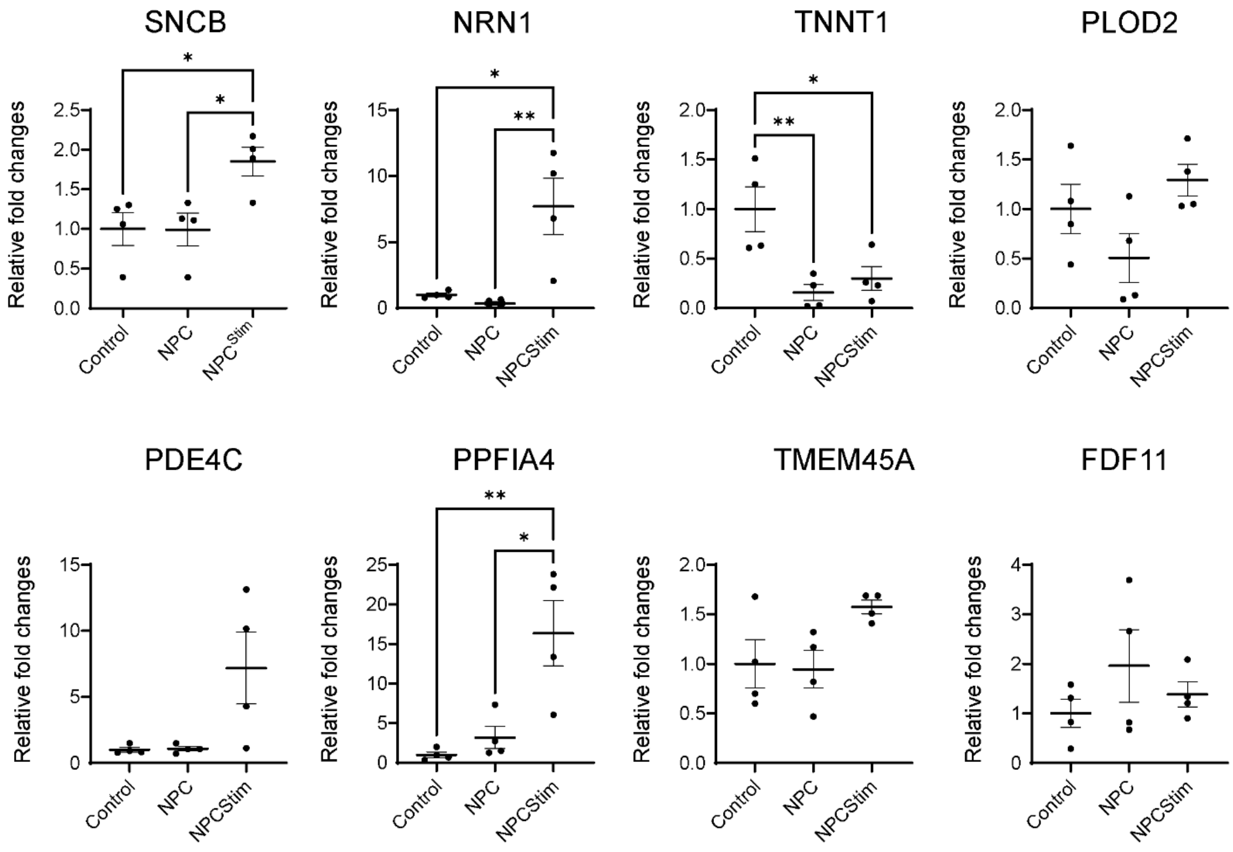

Supplementary Fig. 12 Transcriptome verification with qRT-PCR. qRT-PCR data indicated that electrical stimulation augmented the expression of SNCB, NRN1, TNNT1, PDE4C, PPFIA4, and TMEM45A in NPCs. PLOD2 and FGF11 did not demonstrate augmentation with electrical stimulation. These genes were verified from transcriptome analyses. ** and * indicate statistical significances compared to control and cells with no electrical stimulation (NPC). Analyzed using a one-way ANOVA, followed by Tukey's HSD post hoc test with ** $P<0.01$ and * $P<0.05$, data shown as mean±SEM, n=4.

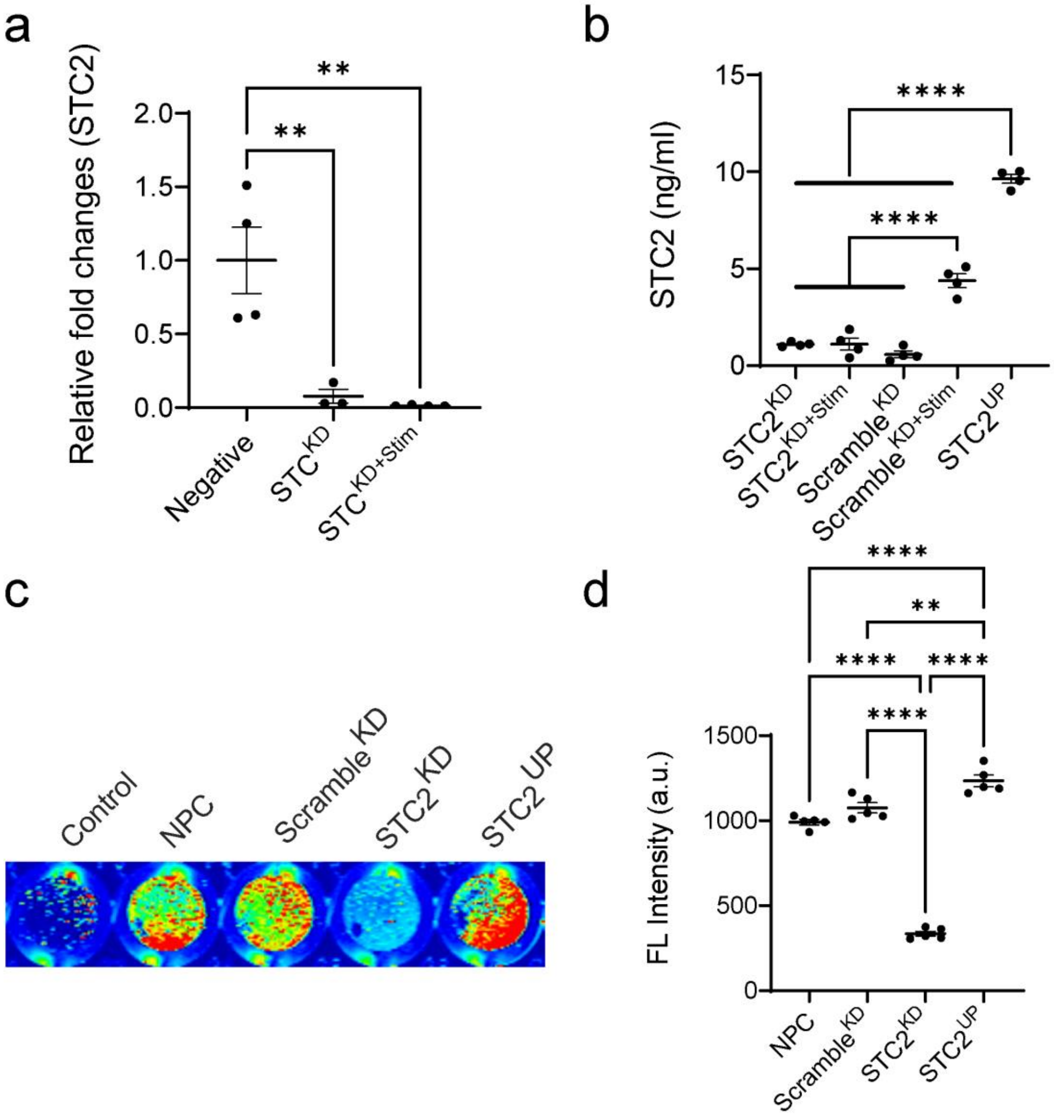

Supplementary Fig. 13 STC2 lentiviral downregulation and upregulation in NPCs *in vitro*. **a,** qRT-PCR data indicated that STC2 expression after knock-down treatment is minimal and electrical stimulation did not accelerate STC2 expression in NPCs. **b,** ELISA indicated that NPCs after STC2 knock-down did not produce STC2, however, the cells after $STC2^{UP}$(lentiviral upregulation of STC2) produced significant amount of STC2 compared to other groups. NPCs receiving a scramble lentivirus show that electrical stimulation upregulates STC2 levels. **c,** Representative in-cell western images of human NPCs in different treatment conditions showing increased STC2 expression in $Scramble^{KD+Stim}$ and $STC2^{UP}$ groups. The color from blue to green to red represents increase in intensity of STC2 levels, whose absolute values are quantified in d. **d,** Quantification of in-cell western images of each group, demonstrating $STC2^{KD}$ reduces STC2 expression. **a,b,d** ** $P<0.01$, **** $P<0.0001$, analyzed using a one-way ANOVA, followed by Tukey's HSD post hoc test, data presented as mean ±SEM, n=4 for a, b; n=5 for c, d.

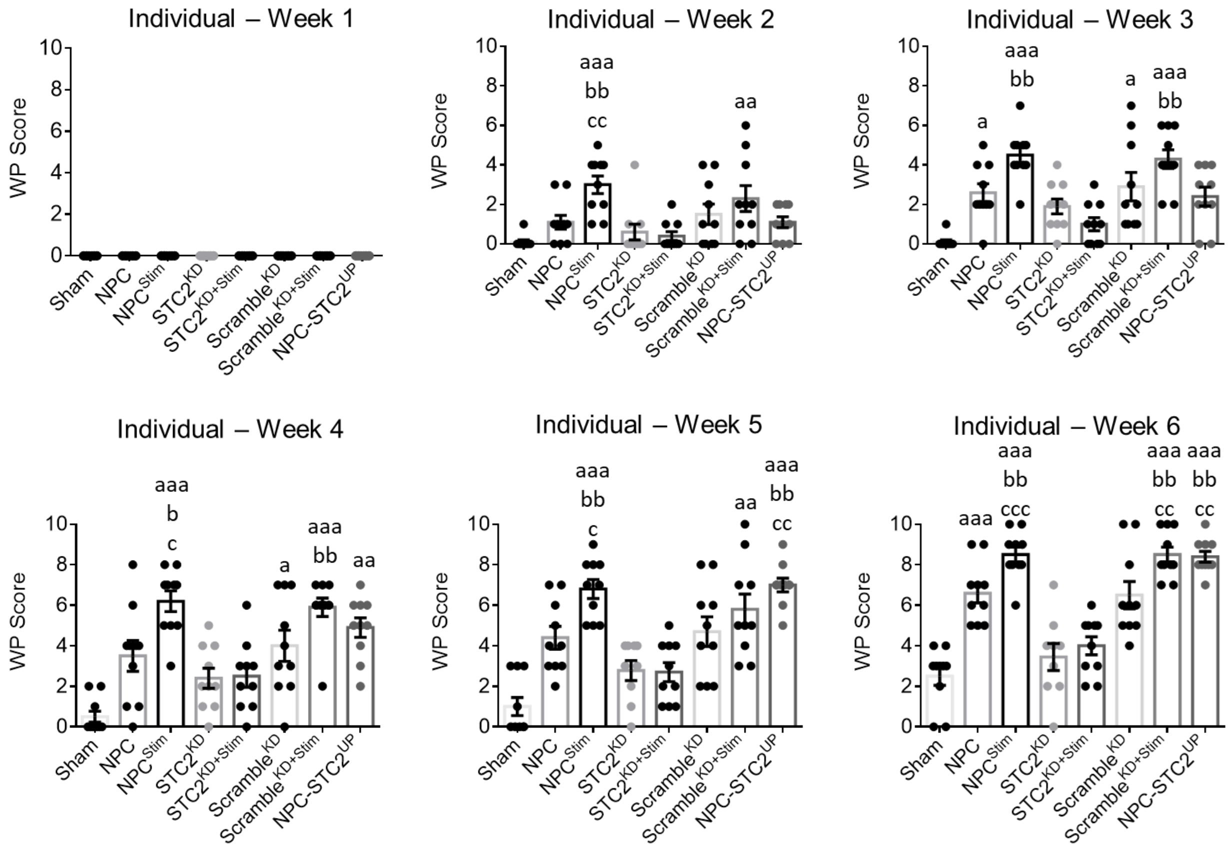

Supplementary Fig. 14 STC2 is an integral component of combined NPC and electrical stimulation functional stroke recovery. Vibrissae-forepaw (WP) behavioral testing for individual weeks. a - indicates statistically significant difference compared to Sham control, b – indicates significant difference compared to $STC2^{KD+Stim}$, and c – indicates significant difference from $STC2^{KD}$. Analyzed using Kruskal-Wallis followed by post hoc pairwise Mann-Whitney tests with Benjamini-Hochberg correction to control the false discovery rate at the 0.05 level; a, b, c = $P<0.05$; aa, bb, cc = $P<0.01$, and aaa, ccc = $P<0.001$ respectively, data shown as mean±SEM, n=10 per group.

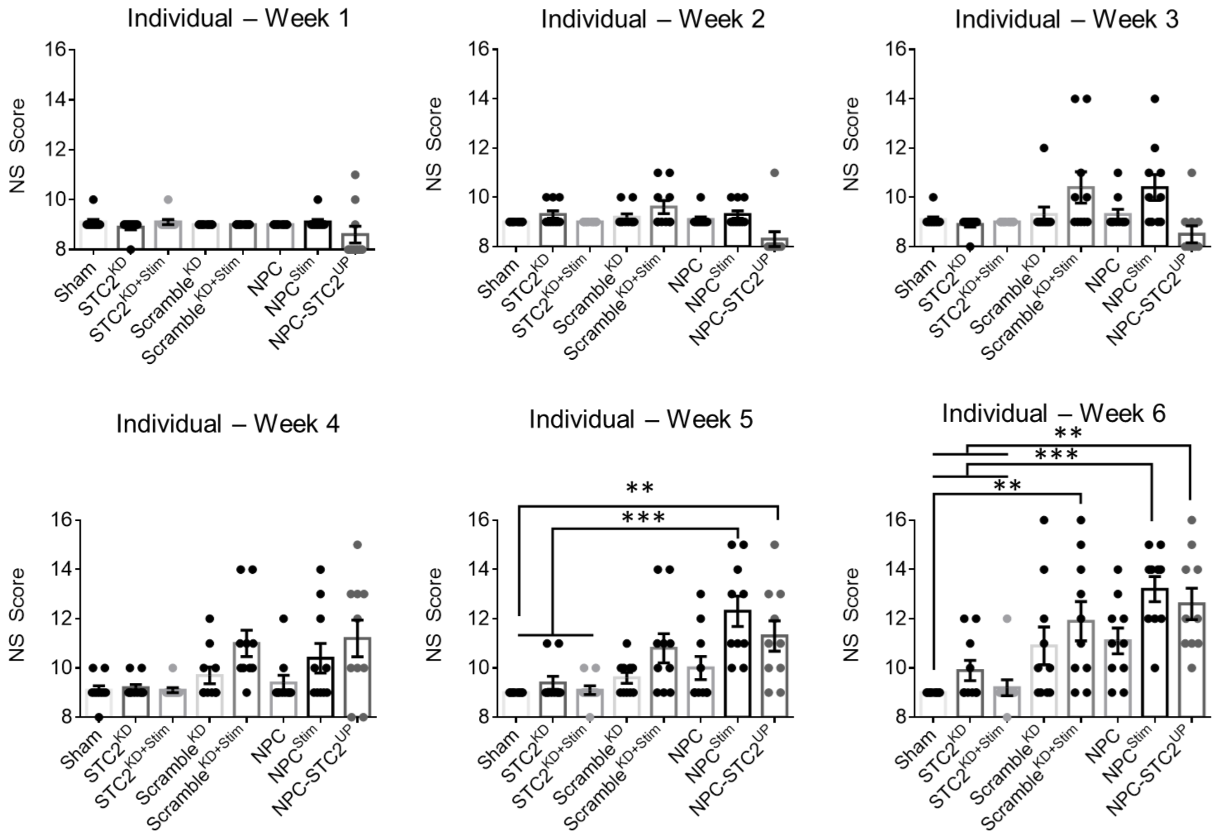

Supplementary Fig. 15 Total neurological score (NS) testing. Neurological score (NS) behavioral testing for individual weeks. Scramble$^{KD+Stim}$, NPC^{Stim} and STC2^{UP}exhibited greater recovery beginning at 4 weeks post-stroke compared to other groups. Analyzed using Kruskal-Wallis followed by post hoc pairwise Mann-Whitney tests with Benjamini-Hochberg correction to control the false discovery rate at the 0.05 level; * $p< 0.05$, ** $p < 0.01$, *** $p<0.001$, data shown as mean±SEM, n=10 per group.

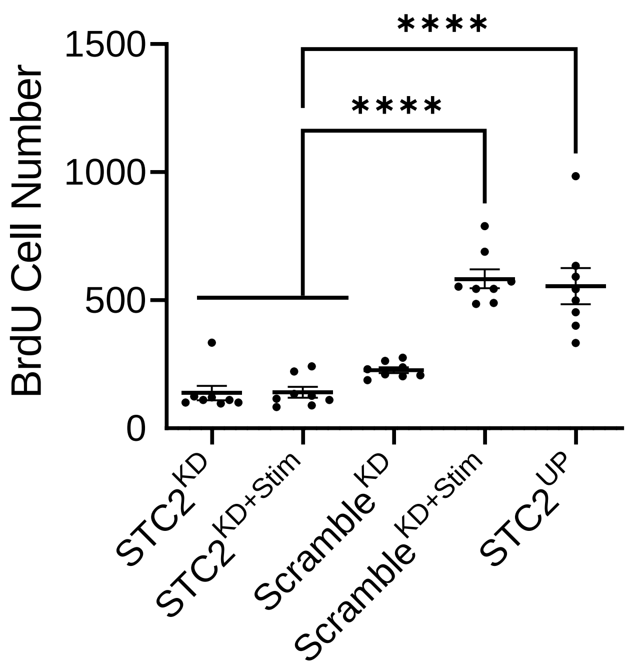

Supplementary Fig. 16 Cell count analysis of post-stroke brain slices. BrdU$^+$ cells in the MD region (between subventricular zone and peri-infarct) for various experimental groups. Analyzed using one-way ANOVA followed by Tukey's HSD post hoc test for multiple comparisons, **** indicates p<0.0001, data shown as mean±SEM, n=8 images/4 rats.

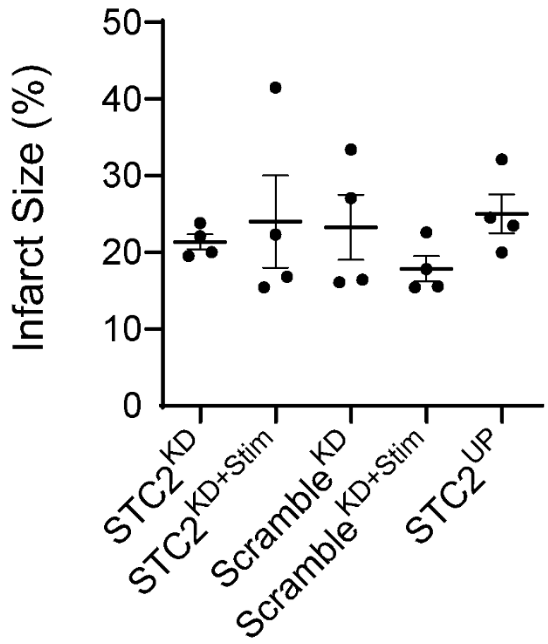

Supplementary Fig. 17 Final infarct size. Percentage calculation of infarct size for $STC2^{KD}$, $STC2^{KD+Stim}$, $Scramble^{KD}$, $Scramble^{KD+Stim}$, and $STC2^{UP}$. There were no statistically significant differences between any group. Analyzed using one-way ANOVA followed by Tukey's HSD post hoc test for multiple comparisons, data shown as mean±SEM, n=4.

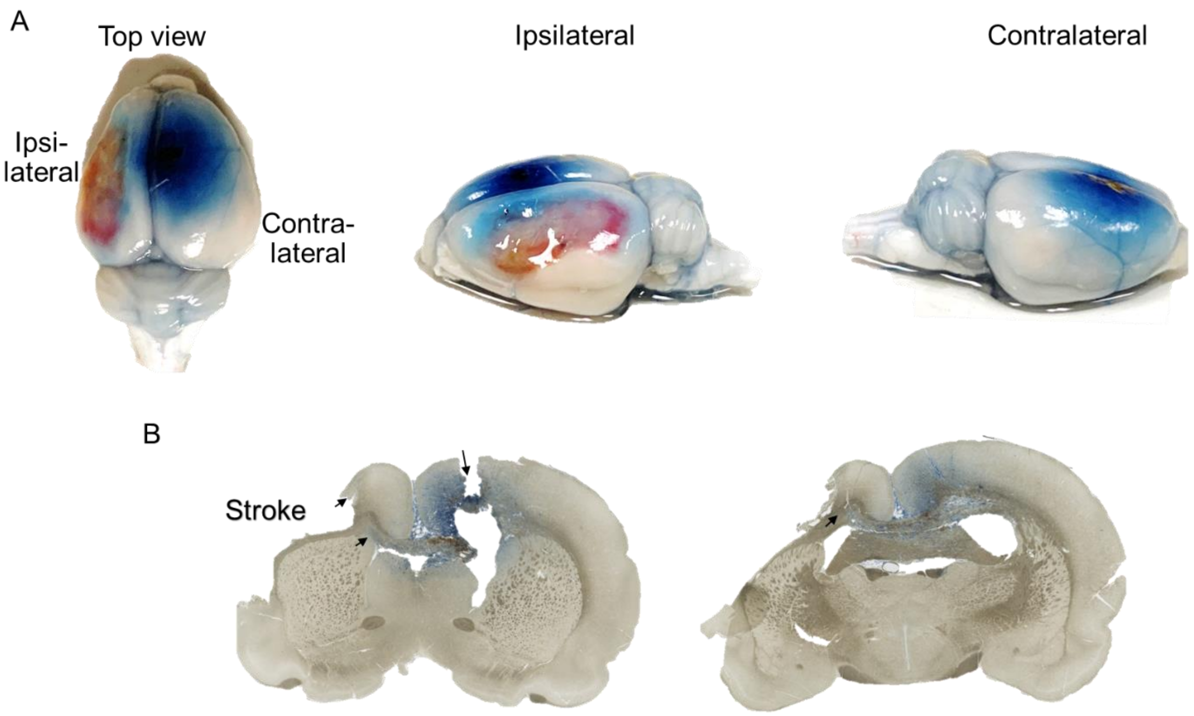

Supplementary Fig. 18 Intraventricular delivery of trypan blue solution via mini-osmotic pump. a, Top and side view of rat brain administered with trypan blue solution after stroke. **b,** Bright field images of brain slices with arrows showing the tear due to needle injected from mini-osmotic pump and trypan blue (blue) distributed from the contra-lateral ventricles towards the subventricular and peri-infarct region.

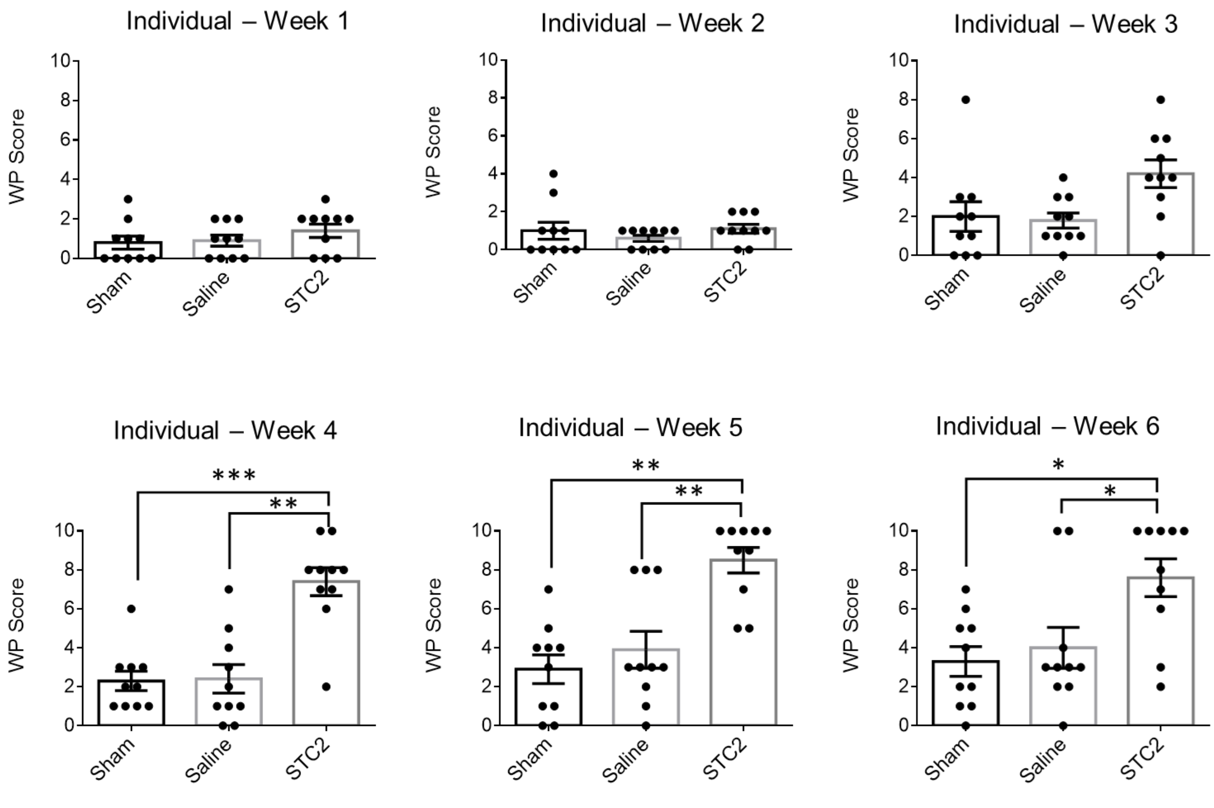

Supplementary Fig. 19 Intraventricular STC2 delivery improves functional stroke recovery. Vibrissae-forepaw (WP) behavioral testing for individual weeks. There was a statistically significant difference between WP Scores of the STC2 group versus both the Sham and Saline group starting at week 4 post-stroke. Analyzed using log-transform two-way repeated measures ANOVA followed by Dunnett's multiple comparisons test, * $p< 0.05$, ** $p < 0.01$, *** $p<0.001$, data shown as mean±SEM, n=10 per group.

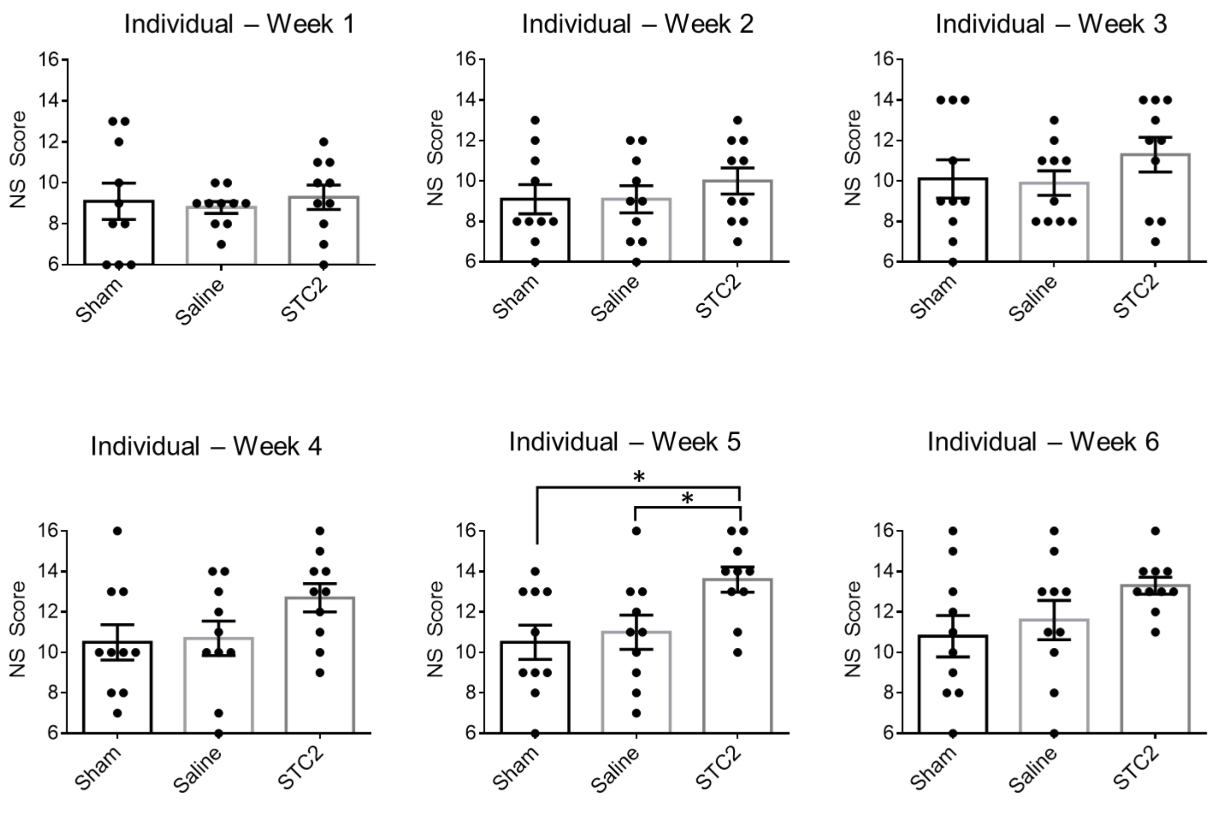

Supplementary Fig. 20 Total neurological score (NS) testing. Neurological score (NS) behavioral testing for individual weeks. There was a statistically significant difference in NS Scores between the STC2 group versus both the Sham and Saline group at 5-weeks post-stroke. Analyzed using Kruskal-Wallis followed by Dunn's multiple comparisons test, * $p < 0.05$, data shown as mean±SEM, n=10 per group.

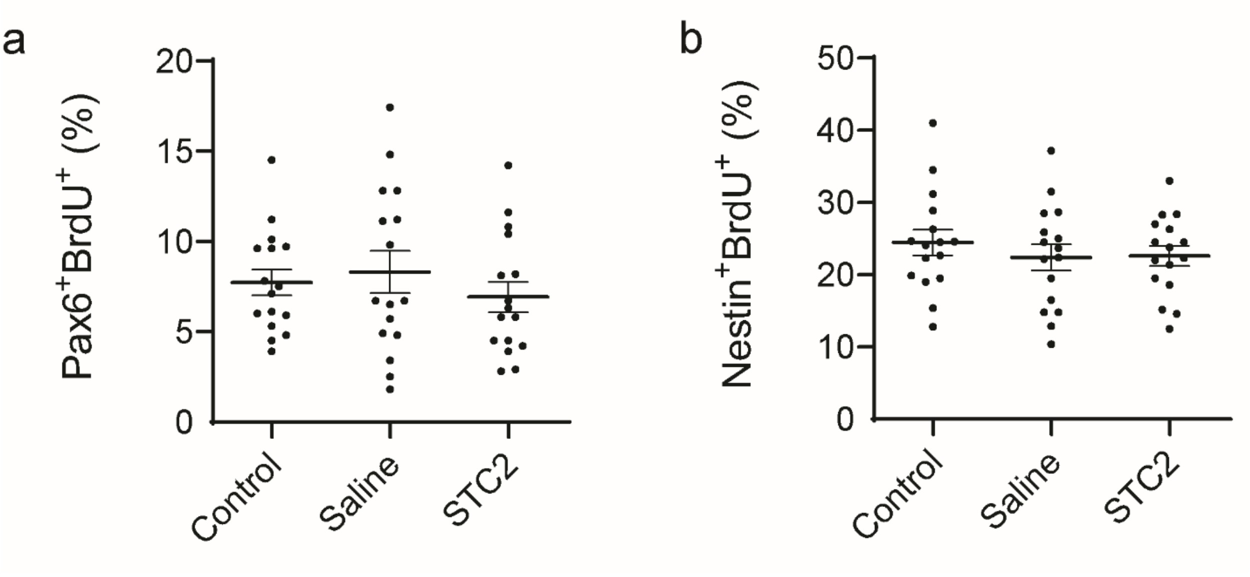

Supplementary Fig. 21 Cell count analysis of co-labeled post-stroke brain slices. a, Cell counts of Pax6$^+$BrdU$^+$; **b,** Nestin$^+$BrdU$^+$ cells in the peri-infarct (PI) tissue. There were no statistically significant differences between any group. Analyzed using a one-way ANOVA, followed by Tukey's HSD post hoc test, data shown as mean±SEM, n=16 images/4 rats.

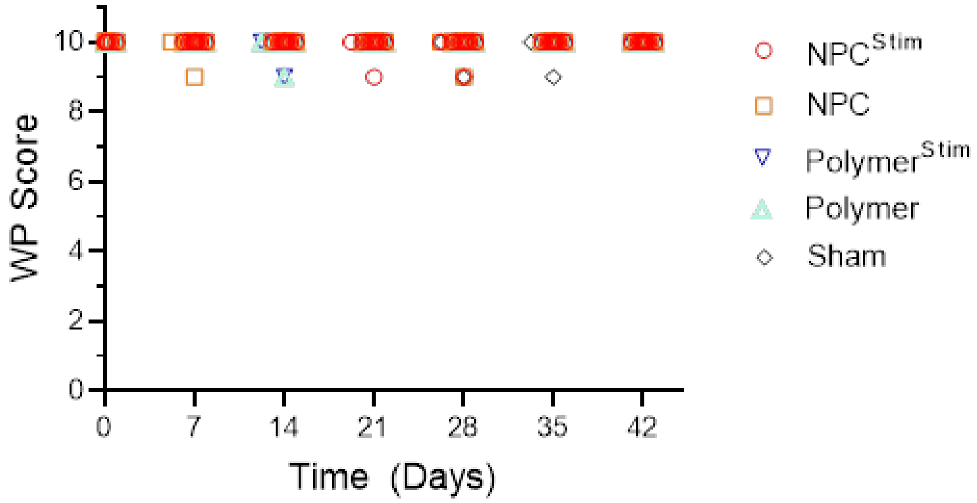

Supplementary Fig. 22 Behavioral assessment scores of the ipsilateral limb (non-effected limb). The vibrissae-forepaw behavioral assay was evaluated for the potential confound of testing effect via longitudinal assessments of the limb ipsilateral to the stroke as a non-effected control. There was no significant difference between WP Scores in any group at any timepoint. Analyzed using Kruskal-Wallis with Dunn's multiple comparisons test, data shown as mean±SEM, n=10 for each group respectively.

Supplementary Table 1. Ingenuity pathway analysis molecules

Top Analysis-Ready Molecules		Expression Value (LogFC)
**Upregulated**	CA9	9.661
	PDE4C	8.106
	PPFIA4	7.997
	AHNAK2	7.728
	SNCB	7.577
	ARFGEF3	7.539
	HSF4	7.391
	NRN1	7.178
	SMIM24	7.070
	STC2	6.905
**Downregulated**	TM4SF18	-3.484
	F13A1	-3.297
	KCNJ6	-2.983
	HES5	-2.706
	SFXN2	-2.564
	ACAT2	-2.529
	SLITRK1	-2.450
	KLF15	-2425
	MYB	-2.403
	RRM2	-2.305

Supplementary Table 2. Top 15 hallmark pathways (blue highlight indicates STC2 is leading edge)

pathway	pval	padj	ES	NES	nMoreExtreme	size	leadingEdge
**HALLMARK_HYPOXIA**	**0.0002155**	**0.0021614**	**0.7928585**	**2.747115**	**0**	**157**	**PPFIA4, STC2, P4HA2, INHA, NDRG1, BHLHE40, ALDOC, AK4, TMEM45A, DDIT4, ANGPTL4, PYGM, FAM162A, RORA, VEGFA, STC1, PPP1R3C, LOX, EFNA1, HMOX1, PFKFB3, P4HA1, CXCR4, PDK1, HK2, ADORA2B, ENO2, PGK1, GBE1, ADM, AMPD3, FOSL2, ALDOA, DDIT3, PTRF, ATF3, KDM3A, PFKP, ANKZF1, ENO1, MXI1, WSB1, SLC2A3, EFNA3, LDHA, NFIL3, GYS1, ANXA2, PNRC1, CA12, PGM1, KIF5A, PLIN2, PPP1R15A, ATP7A, BNIP3L, CP, HK1, GAPDH, KLHL24, NR3C1, RRAGD, ZFP36, DTNA, FOS, GALK1, IRS2, JUN, MIF, PPARGC1A, PDK3, CAV1, TPI1, JMJD6, TNFAIP3, SCARB1, MAFF, BTG1, KDELR3, SERPINE1, SIAH2, TIPARP, IDS, CDKN1C, PDGFB, ENO3, HEXA, CCNG2, VLDLR, FOXO3, DUSP1, GLRX, CITED2, GAA, PGM2, SLC2A1, CDKN1A, CHST3, GPI, NEDD4L, SLC6A6, TPST2**
HALLMARK_E2F_TARGETS	0.0035714	0.0106375	-0.7731125	-3.723593	0	196	RRM2, TRIP13, RFC3, LMNB1, SPC25, DLGAP5, MCM4, CENPE, PLK4, GINS1, HMGB3, KIF4A, TMPO, DSCC1, BUB1B, SMC4, CDC25A, H2AFX, SRSF2, GINS4, SRSF1, MCM6, MYBL2, POLA2, DIAPH3, CENPM, GINS3, AURKB, RAD51AP1, HELLS, MCM5, RANBP1, SUV39H1, MAD2L1, UBE2T, NME1, KIF18B, BIRC5, DEPDC1, CDC20, ASF1B, CSE1L, SPAG5, PRIM2, CHEK1, TACC3, ATAD2, MCM2, AURKA, MELK, BRCA1, CDK1, MCM CKS1B, MCM3, CCNB2, MMS22L, PLK1, BARD1, KIF22, CDCA8, NASP, CCNE1, LIG1, RACGAP1, TOP2A, CDCA3, DEK, MSH2, MKI67, HNRNPD, RNASEH2A, HMMR, KPNA2, USP1, PSMC3IP, H2AFZ, TCF19, TIMELES RPA3, KIF2C, TRA2B, ORC6, PCNA, ESPL1, NUP107, DCTPP1, TIPIN, CDKN2C, NBN, TK1, DNMT1, UNG, EZH2, ING3, POLD1, BRCA2, CBX5, ANP32E, STAG1, POLD3, DUT, UBR7, CDKN3, CIT, SPC24, SMC1A, LBR, LYAR, PHF5A, STMN1, RPA2, SNRPB, RAD1, MXD3, EXOSC8, RFC2, UBE2S, TUBB, TUBG1, PRKDC, SMC6, MLH1, NOP56, ZW10, NUP205, SHMT1, CHEK2, CTCF, CDC25B, DDX39A, PRPS1, PDS5B, POLE, PTTG1, DONSON, PSIP1, E2F8, POP7, HMGB2, RAD51C, ORC2, RAD21, PA2G4, RPA1, RBBP7, NAA38, POLD2, CKS2, EED, WDR90, ILF3, SSRP1, DCK, NUDT21
HALLMARK_G2M_CHECKPOINT	0.0035336	0.0106375	-0.7172888	-3.443899	0	195	CDC7, CDC45, LMNB1, RAD54L, CDC6, CENPE, PLK4, HMGB3, CCNA2, KIF4A, TMPO, KIF15, SMC4, CDC25A, H2AFX, SRSF2, SRSF1, MCM6, EXO1, MYBL2, POLA2, HSPA8, TTK, PBK, AURKB, MCM5, GINS2, PRC1, BUB1, SUV39H1, MAD2L1, NDC80, KIF23, BIRC5, STIL, KIF11, E2F1, CDC20, TPX2, CHAF1A, PRIM2, CCNF, CHEK1, TACC3, UBE2C, MCM2, NUSAP1, AURKA, CENPA, SFPQ, SMC2, CDK1, CKS1B, E2F2, MCM3, CCNB2, PLK1, BARD1, KIF22, CENPF, NASP, RACGAP1, INCENP, TOP2A, MKI67, HNRNPD, HIST1H2BK, FBXO5, HMMR, DBF4, KPNA2, RBL1, POLQ, KIF20B, H2AFZ, HMGN2, SQLE, SNRPD1, AMD1, KIF2C, DTYMK, TRA2B, ORC6, ESPL1, TROAP, CDKN2C, TFDP1, TRAIP, DKC1, EZH2, H2AFV, BRCA2, STAG1, BUB3, CDKN3, SMC1A, LBR, STMN1, RPA2, WRN, LIG3, CCND1, CASP8AP2, E2F3, UBE2S, MARCKS, FANCC, SRSF10, NEK2, CDC27, RBM14, UCK2, CTCF, CDC25B, DDX39A, PDS5B, POLE, EWSR1, PTTG1, NUP50, E2F4
HALLMARK_ANGIOGENESIS	0.0091586	0.0224385	0.6582443	1.715509	31	23	VEGFA, STC1, JAG2, MSX1, JAG1
HALLMARK_MYC_TARGETS_V2	0.0010267	0.0055898	-0.6087484	-2.377404	0	56	MCM4, PLK4, MCM5, UTP20, PLK1, SRM, TMEM97, DCTPP1, GRWD1, RRP9, UNG, MRTO4, PUS1, PRMT3, PPRC1, LAS1L, BYSL, NOP56, HSPE1, NIP7, CBX3, MYBBP1A, NDUFAF4, TCOF1, NOP16, GNL3, RRP12, PA2G4, NOP2, RABEPK, SORD, CDK4, WDR43, SLC19A1, DDX18, HSPD1, NOLC1, NOC4L, TFB2M, AIMP2
HALLMARK_TNFA_SIGNALING_VIA_NFKB	0.0002179	0.0021614	0.6055749	2.079819	0	140	BHLHE40, VEGFA, EFNA1, PFKFB3, KLF10, FOSL2, ATF3, PTPRE, SLC2A3, SQSTM1, TNIP1, CEBPB, NAMPT, NFIL3, PNRC1, PPP1R15A, NR4A3, KDM6B, ZFP36, FOS, BCL6, GADD45B, IRS2, JUN, MXD1, GADD45A, HBEGF, GEM, PER1, EIF1, TNFAIP3, JAG1, MAFF, BTG1, SAT1, TAP1, SERPINE1, SLC16A6, CEBPD, DUSP5, TIPARP, LIF, F2RL1, NFKB2, MAP3K8, DUSP1, ICAM1, NFAT5, RELB, RELA, JUNB, TRIB1, B4GALT1, RHOE CDKN1A, TRIP10, SDC4, PLK2, PHLDA2, NR4A1, KLF2, RNF19B, MYC
**HALLMARK_GLYCOLYSIS**	**0.0002145**	**0.0021614**	**0.5988552**	**2.084232**	**0**	**167**	**PPFIA4, STC2, EGLN3, SLC16A3, P4HA2, AK4, PLOD2, DDIT4, ANGPTL4, SPAG4, FAM162A, VEGFA, STC1, P4HA1, CXCR4, HK2, NOL3, ADORA2B, ENO2, PGK1, ANG, ALDOA, PLOD1, PFKP, ANKZF1, ENO MXI1, EFNA3, LDHA, GYS1, LHPP, RRAGD, GALK1, IRS2, MIF, MPI, DSC2, PDK3, TPI1**
**HALLMARK_MYOGENESIS**	**0.0002206**	**0.0021614**	**0.5945788**	**2.024074**	**0**	**128**	**PPFIA4, STC2, BHLHE40, PYGM, PPP1R3C, TNNT1, TNNC1, KLF5, ERBB3, ACHE, COL1A1, RYR1, HRC, SH3BGR, MEF2C, GSN, ATP2A1, COL6A2, NCAM1, FOXO4, DTNA, GADD45B, HBEGF, MAPRE3, SPEG CD36, EPHB3, HDAC5, OCEL1, TCAP, PTGIS, CHRNB1, CTF1, MEF2D, COL4A2, SIRT2, AK1, ENO3, RIT1, ADCY9, CACNA1H, FKBP1B, KCNH1, TPM2, NQO1, GAA, CDKN1A, KCNH2, KIFC3, PTP4A3**
HALLMARK_P53_PATHWAY	0.0002157	0.0021614	0.5839886	2.022314	0	155	NDRG1, EPS8L2, DDIT4, FAM162A, S100A10, HMOX1, RHBDF2, OSGIN1, DDIT3, ATF3, PTPRE, TRIB3, STOM, TXNIP, JAG2, PPP1R15A, SLC3A2, FOS, JUN, MXD1, GADD45A, SLC7A11, HBEGF, UPP1, BTG1, ABHD4, SAT1, TAP1, VWA5A, BLCAP, VAMP8, ANKRA2, CEBPA, AK1, LIF, CTSD, SERTAD3, TSPYL2, GM2A, FOXO3, HIST3H2A, H2AFJ, CDKN1A, MKNK2, MAPKAPK3, PLK2, PLK3, ZFP36L1, RALGDS, RNF19B, ACVR1B, HEXIM1, ZMAT3, TOB1, PMM1, HSPA4L
HALLMARK_KRAS_SIGNALING_DN	0.0004813	0.0033694	0.5736759	1.805565	1	69	SNCB, SLC6A3, MYO15A, ARHGDIG, IGFBP2, KCNN1, P2RX6, RGS11, SLC38A3, KCND1, TNNI3, RYR1, YBX2, DLK2, CLSTN3, COPZ2, SLC29A3
HALLMARK_MYC_TARGETS_V1	0.0035714	0.0106375	-0.5627247	-2.710288	0	196	CDC45, MCM4, CCNA2, SRSF7, RRM1, SRSF2, SRSF1, MCM6, MCM5, RANBP1, MAD2L1, NME1, HNRNPA2B1, CDC20, MCM2, MCM7, DEK, HNRNPD, TYMS, KPNA2, RFC4, USP1, SRSF3, SRM, H2AFZ, SNRPD1, NCBP1, CCT5, TRA2B, PCNA, ABCE1, HNRNPR, TFDP1, RRP9, FAM120A, CSTF2, PSMD1, DUT, BUB3, PRDX3, SRPK1, PRPS2, SNRPA1, HNRNPA3, SNRPG, PSMB2, PSMD14, TARDBP, ILF2, C1QBP, CDK2, NOPS6 HSPE1, CBX3, GLO1, SNRPA, EIF4G2, EIF4E, ERH, MRPL23, PPIA, NHP2, VBP1, GOT2, NOP16, GNL3, PSMB3, DHX15, ORC2, DDX21, HNRNPU, PA2G4, SNRPD3, POLD2, POLE3, YWHAQ, MRPL9, PSMA4, UBA2, EIF3J, YWHAE, SSBP1, CCT2, KPNB1, RAN, STARD7, CDK4, SERBP1, PPM1G, SET, EIF1AX, SMARCC1, XRCC6, DDX18, EIF2S1, G3BP1, CUL1, ACP1, CCT7, HSPD1, NOLC1, HDGF, HDAC2, LSM7, ETF1, CCT3, PSMA AIMP2, PRPF31, SYNCRIP, HDDC2, NCBP2, PHB, SF3B3, HNRNPC, SSB, EEF1B2, PSMD3, SNRPB2
HALLMARK_COAGULATION	0.0110710	0.0258323	0.5028967	1.582797	45	69	ANG, CD9, F12, ITGA2, GSN, CTSK, LRP1, C1R, F10, MST1, MAFF, SERPINE1, SIRT2, CTSB, PDGFB, LTA4H, F8, MMP11, LGMN, ANXA1, KLF7, LAMP2, CTSH, C1S, SH2B2, FURIN, F2RL2
HALLMARK_HEME_METABOLISM	0.0004342	0.0033694	0.4886425	1.684929	1	146	P4HA2, TNS1, OPTN, BLVRB, MXI1, PPP2R5B, ASNS, RAP1GAP, HTATIP2, BNIP3L, NR3C1, RIOK3, SLC7A11, EPOR, SIDT2, ISCA1, SMOX, TFRC, ELL2, BCAM, CTSB, SLC6A9, ENDOD1, ATP6V0A1, YPEL5, FN3K, FOXO3, NFE2L1, ATG4A, LRP10, NARF, SLC2A1, SNCA, H1F0, MARCH8, EZH1, CLCN3, LAMP2, KHNYN, SLC10A3, MKRN1, HEBP1, SDCBP, GYPC, CAST, ALAD, CTNS, PGLS, BTG2, RBM38, MARCH2, TSPAN5, NEK7 SYNJ1, SLC6A8, IGSF3, AGPAT4, FBXO34, TMCC2, CCDC28A, MGST3, RNF19A, GCLE
**HALLMARK_ESTROGEN_RESPONSE_EARLY**	**0.0008770**	**0.0053716**	**0.4743593**	**1.625388**	**3**	**137**	**STC2, BHLHE40, DHRS3, MAPT, KLF10, TTC39A, SEMA3B, BLVRB, SEC14L2, PAPSS2, CA12, DHRS2, SLC7A5, KRT8, FOS, PLA2G16, RARA, KDM4B, SH3BP5, TBC1D30, ALDH3B1, SCARB1, PEX11A, ZNF185, RAPGEFL1, SIAH2, SYNGR1, ENDOD1, TIPARP, ADCY9, UNC119, FLNB, B4GALT1, SLC2A1, TSKU, AFF1, IGF1R, MYC, CALCR, TOB1, ELF1, NRIP1, FAM63A, CELSR1**
HALLMARK_APOPTOSIS	0.0017746	0.0086957	0.4680321	1.586828	7	124	HMOX1, ENO2, DDIT3, ATF3, SMAD7, ERBB3, TXNIP, PPP2R5B, SQSTM1, TSPO, GSN, BNIP3L, HSPB1, RARA, GADD45B, JUN, GADD45A, BMF, CAV1, SAT1, TAP1, LMNA, SATB1, RELA, MADD, IGF2R, NEFH, RHOB, CDKN1A, ANXA1, H1F0, PDGFRB

Supplementary Table 3. Upregulated genes due to electrical stimulation

	RNA Seq			qRT-PCR		
Gene	Log FC	Adjusted p-value	Significance	Mean Diff. between fold change ($\Delta\Delta C_T$) NPC^{stim} and NPC	Adjusted p-value	Significance
STC2	6.9	0.00001	****	12.5	0.003	**
SNCB	7.6	0.00003	****	0.9	0.034	*
NRN1	7.2	0.00034	***	7.3	0.006	**
TNNT1	4.0	0.00004	****	0.1	0.797	ns
PLOD2	5.7	0.00000	****	0.8	0.081	ns
PDE4C	8.1	0.00005	****	6.1	0.055	ns
PPFIA4	8.0	0.00013	***	13.1	0.013	*
TMEM45A	5.0	0.00011	***	0.6	0.087	ns
FGF11	4.7	0.00033	***	0.6	0.681	ns

Supplementary Table 4.

**Modified Neurological Severity Score**	**Points**
Motor	
Postural Reflux	2
2 symmetric touchdown (normal)	
1 asymmetric touchdown + resistant to pushing	
0 asymmetric touchdown + no resistance to pushing	
Raising rat from the base of tail	3
3 Both paws touching surface simultaneously	
2 One paw touches the surface before the other	
1 Flexion of forelimb but unable to touch	
0 Unable to touch any limb (forelimb or hindlimb)	
	3
Walking	
3 Normal	
2 Difficulty walk straight	
1 Circling in the direction of the paretic side	
0 Falling to paretic side	2
Sensory	
2 Proprioceptive test (pressing paw against table edge to stimulate muscles)	
1 Placing test (responds to visual and tactile stimuli)	
Beam Test	6
6 Balances with steady posture and crosses beam without any paw slip	
5 Grasps side of beam without slipping	
4 Hugs the beam and one limb slips from the beam	
3 Hugs the beam and two limbs fall down from the beam	
2 Attempts to balance on the beam but falls off (>40s)	
1 Attempts to balance on the beam but falls off (>60s)	

0 Falls off without attempt to stay on (<20s)	
Maximum Points	16

Supplementary References

1 George, P. M. *et al.* Electrical preconditioning of stem cells with a conductive polymer scaffold enhances stroke recovery. *Biomaterials* **142**, 31-40, doi:10.1016/j.biomaterials.2017.07.020 (2017).
