# Supplementary material for: Electrical modulation of transplanted stem cells improves functional recovery in a rodent model of stroke
Source: Nat Commun. 2022 Mar 15;13:1366. doi: 10.1038/s41467-022-29017-w (PMC8924243; doi:10.1038/s41467-022-29017-w)
Supplement: Supplementary file 2 — Reporting Summary [file 41467_2022_29017_MOESM2_ESM.pdf]

nature portfolio

Corresponding author(s): Paul M. George, M.D., Ph.D.

Last updated by author(s): January 31, 2022

Reporting Summary

Nature Portfolio wishes to improve the reproducibility of the work that we publish. This form provides structure for consistency and transparency in reporting. For further information on Nature Portfolio policies, see our Editorial Policies and the Editorial Policy Checklist.

Statistics

For all statistical analyses, confirm that the following items are present in the figure legend, table legend, main text, or Methods section.

n/a	Confirmed	
☐	☒	The exact sample size (n) for each experimental group/condition, given as a discrete number and unit of measurement
☐	☒	A statement on whether measurements were taken from distinct samples or whether the same sample was measured repeatedly
☐	☒	The statistical test(s) used AND whether they are one- or two-sided<br>*Only common tests should be described solely by name; describe more complex techniques in the Methods section.*
☐	☒	A description of all covariates tested
☐	☒	A description of any assumptions or corrections, such as tests of normality and adjustment for multiple comparisons
☐	☒	A full description of the statistical parameters including central tendency (e.g. means) or other basic estimates (e.g. regression coefficient) AND variation (e.g. standard deviation) or associated estimates of uncertainty (e.g. confidence intervals)
☐	☒	For null hypothesis testing, the test statistic (e.g. F, t, r) with confidence intervals, effect sizes, degrees of freedom and P value noted<br>*Give P values as exact values whenever suitable.*
☒	☐	For Bayesian analysis, information on the choice of priors and Markov chain Monte Carlo settings
☒	☐	For hierarchical and complex designs, identification of the appropriate level for tests and full reporting of outcomes
☒	☐	Estimates of effect sizes (e.g. Cohen's d, Pearson's r), indicating how they were calculated

Our web collection on statistics for biologists contains articles on many of the points above.

Software and code

Policy information about availability of computer code

Data collection	Odyssey CLx Imaging Studio 3.1 Analysis Software (Licor)
Data analysis	GraphPad Prism 9.2.0; ImageJ 1.52a (National Institutes of Health)

For manuscripts utilizing custom algorithms or software that are central to the research but not yet described in published literature, software must be made available to editors and reviewers. We strongly encourage code deposition in a community repository (e.g. GitHub). See the Nature Portfolio guidelines for submitting code & software for further information.

Data

Policy information about availability of data

All manuscripts must include a data availability statement. This statement should provide the following information, where applicable:

- Accession codes, unique identifiers, or web links for publicly available datasets
- A description of any restrictions on data availability
- For clinical datasets or third party data, please ensure that the statement adheres to our policy

All data are available in the article and supplementary materials. RNA sequencing data has been submitted to the Gene Expression Omnibus (GEO accession #GSE185402). Additional data is available from the corresponding author upon reasonable request.

Field-specific reporting

Please select the one below that is the best fit for your research. If you are not sure, read the appropriate sections before making your selection.

☒ Life sciences ☐ Behavioural & social sciences ☐ Ecological, evolutionary & environmental sciences

For a reference copy of the document with all sections, see nature.com/documents/nr-reporting-summary-flat.pdf

Life sciences study design

All studies must disclose on these points even when the disclosure is negative.

Sample size	Sample size was determined based on our previous report: George, P.M. et al. Electrical preconditioning of stem cells with a conductive polymer scaffold enhances stroke recovery. Biomaterials 142, 31-40.
Data exclusions	All excluded data is explained in the methods section.
Replication	Experiments were performed independently according to sample size calculations; additional replication beyond the data reported was not conducted.
Randomization	This study does not involve clinical research participants. All samples/rodents were allocated into experimental groups at random.
Blinding	Investigators were blinded to group allocations during data collection and analyses for all experimentation, including in vitro, in vivo, and behavioral studies.

Reporting for specific materials, systems and methods

We require information from authors about some types of materials, experimental systems and methods used in many studies. Here, indicate whether each material, system or method listed is relevant to your study. If you are not sure if a list item applies to your research, read the appropriate section before selecting a response.

Materials & experimental systems

n/a	Involved in the study	
☐	☒	Antibodies
☐	☒	Eukaryotic cell lines
☒	☐	Palaeontology and archaeology
☐	☒	Animals and other organisms
☒	☐	Human research participants
☒	☐	Clinical data
☒	☐	Dual use research of concern

Methods

n/a	Involved in the study	
☒	☐	ChIP-seq
☒	☐	Flow cytometry
☒	☐	MRI-based neuroimaging

Antibodies

Antibodies used	Primary Antibodies: anti-BrdU (1:20, Abcam, Cat. #AB92837); anti-GFAP (1:1000, Millipore Sigma, Cat. #AB5804); anti-Pax6 (1:100, Fisher Scientific, Cat. #MAB5552MI); anti-NeuN (1:500, Cell Signaling Technology, Cat. #12943); anti-Nestin (1:200, Millipore Sigma, Cat. #ABD69MI); anti-PECAM (1:100, Millipore Sigma, Cat. #MAB1393); anti-TUJ1 (1:100, Neuromics, Cat. #MO15013).<br>Secondary Antibodies: Goat anti-Rabbit IgG AlexaFluor 488 (1:500, Thermofisher Scientific, Cat. #A-11008); Goat anti-Mouse IgG AlexaFluor 488 (1:500, Thermofisher Scientific, Cat. #A11029); Goat anti-Chicken IgY AlexaFluor 555 (1:500, Thermofisher Scientific, Cat. #A-21437); Goat anti-Mouse IgG AlexaFluor 647 (1:500, Thermofisher Scientific, Cat. #A32728).
Validation	anti-BrdU (1:200, Abcam): Cao, X et al. (2020) Apoptosis 25:341-353<br>anti-GFAP (1:1000, Millipore Sigma): Lim, S et al. (2015) Nature Communications 6,8244<br>anti-Pax6 (1:100, Fisher Scientific): Bassett, E et al. (2016) Elife Nov8;5:e16764<br>anti-NeuN (1:500, Cell Signaling Technology): Mullen, R.J. et al. (1992) Development 116,201-11<br>anti-Nestin (1:200, Millipore Sigma): Zou, H et al. (2015) Scientific Reports 5:8468<br>anti-PECAM (1:100, Millipore Sigma): Li, Y e tal. (2018) Frontiers in physiology 9:527<br>anti-TUJ1 (1:100, Neuromics): Corns, LF (2015) Stem Cells 9:2864-76

Eukaryotic cell lines

Policy information about cell lines

Cell line source(s)	hNP1 Aruna Biomedical

Authentication	The cell line was authenticated per the specifications of the vendor. The cell line was not authenticated by our lab.
Mycoplasma contamination	The cell line was tested for mycoplasma contamination per the specifications of the vendor. The cell line was not tested for mycoplasma concentration by our lab.
Commonly misidentified lines<br>(See ICLAC register)	No commonly misidentified lines were used in this study.

Animals and other organisms

Policy information about studies involving animals; ARRIVE guidelines recommended for reporting animal research

Laboratory animals	Adult, male T-cell deficient nude rats, 7-8 weeks old (NIH-RNU)<br>Adult, male Sprague Dawley rats, 7-8 weeks old (Charles River)
Wild animals	No wild animals were used in this study.
Field-collected samples	No field-collected samples were used in this study.
Ethics oversight	All animal procedures were approved by Stanford University's Administrative Panel on Laboratory Animal Care, APLAC protocol #31909, SCRO #616

Note that full information on the approval of the study protocol must also be provided in the manuscript.
